# Further revision of the genus *Megalopsalis* (Opiliones, Neopilionidae), with the description of seven new species

**DOI:** 10.3897/zookeys.328.5439

**Published:** 2013-09-03

**Authors:** Christopher K. Taylor

**Affiliations:** 1 Dept of Environment and Agriculture, Curtin University of Technology, GPO Box U1987, Perth, WA 6845, Australia

**Keywords:** Taxonomy, harvestmen, Australasia

## Abstract

The Australian harvestmen genus *Megalopsalis* (Neopilionidae: Enantiobuninae) is recognised as a senior synonym of the genera *Spinicrus* and *Hypomegalopsalis*, and seven new species are described in *Megalopsalis*: *Megalopsalis suffugiens*, *Megalopsalis walpolensis*, *Megalopsalis caeruleomontium*, *Megalopsalis atrocidiana*, *Megalopsalis coronata*, *Megalopsalis puerilis* and *Megalopsalis sublucens*. A morphological phylogenetic analysis of the Enantiobuninae is also conducted including the new species. Monophyly of Neopilionidae and Enantiobuninae including ‘Monoscutidae’ is corroborated, with the Australasian taxa as a possible sister clade to the South American *Thrasychirus*.

## Introduction

The Enantiobuninae (previously Monoscutidae–see Taylor 2011) are the most speciose group of long-legged harvestmen (Eupnoi) in Australasia, with over 45 described species (Taylor 2004, 2011). The taxonomy of Australasian Enantiobuninae was long based on a small number of characters of doubtful significance, such as the presence or absence of a distal apophysis on the pedipalpal patella, or a ventral tooth-row on the tarsal claw (Forster 1949). Recent reviews of the genera *Pantopsalis* Simon, 1879 (Taylor 2004) and *Megalopsalis* Roewer, 1923 (Taylor 2011) have refined the definitions of those genera and improved our understanding of enantiobunine systematics; however, the genus *Spinicrus* Forster, 1949 remained unrevised. The current paper completes the revision of genera of Australasian Enantiobuninae.

Forster (1949) established *Spinicrus* with the type species *Pantopsalis tasmanica* Hogg, 1910 from Tasmania, distinguishing it from the New Zealand genus *Pantopsalis* (Hogg, 1910) on the basis of the presence of a ventral tooth-comb on the pedipalpal claw (a character that had been accorded high significance in the artificial classification of Eupnoi used by Roewer 1923). Forster (1949) assigned two new species *Spinicrus camelus* Forster, 1949 and *Spinicrus stewarti* Forster, 1949 to the genus, and suggested that *Pantopsaliscontinentalis* Roewer, 1923 of Queensland might also belong to *Spinicrus*. Hickman (1957) later described two new species from Tasmania, *Spinicrus nigricans* Hickman, 1957 and *Spinicrus thrypticum* Hickman, 1957. Around the same time, Kauri (1954) assigned two species from Western Australia to the genus, *Spinicrus minimum* Kauri, 1954 and *Spinicrus porongorupense* Kauri, 1954.

Despite the small number of included species, *Spinicrus* was a morphologically heterogeneous assemblage right from its initial establishment (Forster 1949). The absence of a pedipalp apophysis (distinguishing it from *Megalopsalis*) and the presence of a toothed pedipalp claw remained the only defining characters of the genus; neither of these characters was unique to *Spinicrus* and both are likely to be plesiomorphic for Enantiobuninae as a whole (Taylor 2011). Taylor and Hunt (2009) separated *Spinicrus camelus* and *Spinicrus continentale* from *Spinicrus* as part of the morphologically distinct genus *Neopantopsalis* Taylor & Hunt, 2009 but did not consider the status of the remaining species. The possibility that some of the species, particularly *Spinicrus nigricans* and the Western Australian species, might also need to be transferred to new genera had previously been raised by Hunt (1990) after examination of their distinct spiracle morphologies. In the phylogenetic analysis of Enantiobuninae by Taylor (2011), *Spinicrus* was not identified as monophyletic. The current paper expands Taylor’s (2011) analysis with the addition of seven new species that would have previously been assigned to *Spinicrus*. *Spinicrus* is identified as paraphyletic with regard to the genera *Megalopsalis* and *Hypomegalopsalis* Taylor, 2011, and all three genera are combined into a single genus *Megalopsalis*.

## Material and methods

Specimens came from the collections of the Australian Museum, Sydney(AMS), Museum Victoria, Melbourne(MV), Queensland Museum, Brisbane(QM) and Western Australian Museum, Perth(WAM). Specimens were examined using a Leica MZ6 microscope, and drawings were made using a camera lucida. Photographs and measurements were taken using a Nikon SMZ1500 stereo microscope and the NIS-Elements D 4.00.03 programme, and a Leica DM2500 compound microscope. Genitalia were retained in a microvial with the original specimen. Colouration described is as preserved in alcohol. Specimens to be photographed by SEM were dried using hexamethyldisilazane(HMDS) as described by Nation (1983). The specimens were then mounted and sputter-coated with gold and examined with a Philips XL30 SEM.

Measurements were taken using a reticle. The number of specimens measured is given as “N = x” at the beginning of each description. Measurements are reported as the mean in millimetres, with total recorded range in parentheses; if no range is given, no variation was recorded. For those species in which not all available specimens were measured, the individuals measured are indicated as such in the specimen listings.

For those species in which discrete male morphs can be identified, separate descriptions are given for each form. The larger and smaller morphs are here referred to as ‘major’ and ‘minor’ males, respectively. Other sources dealing with dimorphic males have referred to the smaller morph as ‘effeminate’ (e.g. Forster 1954); however, this terminology is inappropriate for enantiobunines as both morphs are morphologically distinct from females.

### Key to males of genera of Enantiobuninae

*Thrasychiroides brasilicus* Soares & Soares, 1947 has had to be omitted from the following key, as it has not been redescribed since its original description (Soares and Soares 1947) and most of the characters used in the key remain unknown for it. *Thrasychiroides* is the only genus of Enantiobuninae described from South America other than *Thrasychirus*, from which Soares and Soares (1947) distinguished it by the lack of an apophysis on the pedipalp patella. ‘*Megalopsalis*’ *triascuta* Forster, 1944, whose inclusion in that genus requires investigation (Taylor 2011), is keyed out separately from *Megalopsalis*.

**Table:** 

1	Legs relatively short, femur I less than twice length of prosoma; dorsum of opisthosoma usually conspicuously ornamented	2
–	Legs long, femur I more than twice length of prosoma	5
2	Bristle groups on right side of shaft-glans junction only; stylus conspicuously inflated (eastern Australia)	*Australiscutum*
–	Bristle groups on both sides of shaft-glans junction; stylus not inflated (New Zealand)	3
3	Opisthosoma with large flanking spines on lateral margins of dorsum	*Acihasta salebrosa*
–	Opisthosoma without such large spines	4
4	Dorsal denticles complex, laterally extended with raised lateral lobes; chelicerae small, unarmed	*Monoscutum titirangiense*
–	Dorsal denticles simple, subcircular; chelicerae with second segment swollen, heavily denticulate	*Templar incongruens*
5	Mobile hinge present between leg basitarsus and distitarsus; individual spines as lateral processes on penis (South America)	*Thrasychirus*
–	Junction between basitarsus and distitarsus fused, not hinged; bristle groups as lateral processes on penis (Australasia)	6
6	Pedipalp patella with distinct elongate (longer than broad) prodistal apophysis	7
–	Apophysis on pedipalp patella absent or, if present, not distinctly longer than broad	9
7	Pedipalp patella apophysis much longer than main body of patella (North Island, New Zealand)	‘*Megalopsalis*’ *triascuta*
–	Pedipalp patella apophysis shorter than main body of patella (Australia)	8
8	Chelicerae with distinct frontodistal bulge; glans significantly longer than wide, bent distinctly dorsad from shaft, with vertical platelike lateral process on left side of shaft-glans junction (Western Australia)	*Tercentenarium linnaei*
–	Chelicerae without frontodistal bulge; glans not longer than wide, subtriangular in dorsal view, not bent significantly dorsad from shaft, no platelike lateral process	*Megalopsalis* (in part)
9	Glans in lateral view distinctly short and very deep, about as deep as long (New Zealand)	*Mangatangi parvum*
–	Glans elongate or, if relatively short, then distinctly less deep than long	10
10	Pedipalpal claw with ventral tooth-comb; prolateral margin of pedipalpal patella not hypersetose, lacking apophysis (Australia)	11
–	Pedipalpal claw usually without a tooth-comb; if ventral teeth present, then prolateral margin of pedipalp patella densely hypersetose *or* with small distal apophysis (New Zealand)	12
11	Dorsum of prosoma often raised in humps; proventral row of hypertrophied spines along femur I; glans in ventral view elongate, more than twice as long as wide, oval or oblong (New South Wales, Queensland)	*Neopantopsalis*
–	Dorsum of prosoma never raised in humps; glans in ventral view less than twice as long as wide, more or less subtriangular	*Megalopsalis* (in part)
12	Patella of pedipalp prolaterally densely hypersetose, entirely without apophysis; coxa of pedipalp unarmed	*Pantopsalis*
–	Patella of pedipalp not prolaterally hypersetose, often with small triangular prodistal apophysis; coxa of pedipalp with array of blunt tubercles on median side	*Forsteropsalis*

## Taxonomy

### Megalopsalis

Roewer, 1923

http://species-id.net/wiki/Megalopsalis

Macropsalis Sørensen, 1886: 54–55 *non* Sclater 1866 – Pocock 1903: 398; Hogg 1910: 277; Roewer 1911: 102, 1912: 278.
Megalopsalis Roewer, 1923: 866 (replacement name for *Macropsalis* Sørensen) – Forster 1944: 184–185 (referring to material of *Forsteropsalis* Taylor, 2011); Crawford 1992: 28, 29; Taylor 2011: 31.
Spinicrus Forster, 1949: 63 syn. n.; Hickman 1957: 73; Crawford 1992: 43.
Hypomegalopsalis Taylor, 2011: 45 syn. n.

#### Type species.

*Macropsalis serritarsus* Sørensen, 1886 by monotypy.

#### Other included species.

*Megalopsalis serritarsus*-species group: *Megalopsalis epizephyros* Taylor, 2011, *Megalopsalis eremiotis* Taylor, 2011, *Megalopsalis hoggi* Pocock, 1903, *Megalopsalis pilliga* Taylor, 2011.

*Megalopsalis leptekes*-species group: *Megalopsalis leptekes*, 2011, *Megalopsalis tanisphyros* (Taylor, 2011), comb. n. (=*Hypomegalopsalis tanisphyros*).

*Megalopsalis minima*-species group: *Megalopsalis minima* (Kauri, 1954), comb. n. (=*Spinicrus minimum*), *Megalopsalis porongorupensis* (Kauri, 1954), comb. n. (=*Spinicrus porongorupense*), *Megalopsalis suffugiens* sp. n., *Megalopsalis walpolensis* sp. n..

Species not placed in species groups: *Megalopsalis atrocidiana* sp. n., *Megalopsalis caeruleomontium* sp. n., *Megalopsalis coronata* sp. n., *Megalopsalis puerilis* sp. n., *Megalopsalis stewarti* (Forster, 1949), comb. n. (=*Spinicrus stewarti*), *Megalopsalis sublucens* sp. n., *Megalopsalis tasmanica* (Hogg, 1910), comb. n. (=*Pantopsalis tasmanica*), *Megalopsalis thryptica* (Hickman, 1957), comb. n. (=*Spinicrus thrypticum*).

#### Diagnosis.

*Megalopsalis* can be distinguished from all other genera of Enantiobuninae by its male genital morphology, with the glans being relatively short, broad, distally flattened, and more or less subtriangular in ventral view (e.g. Fig. 3d). It can be further distinguished from *Monoscutum*, *Acihasta*, *Templar* and *Australiscutum* by having the legs relatively long and thin, and the dorsum of the opisthosoma weakly sclerotised and unarmed (except *Megalopsalis atrocidiana*; Forster 1948; Taylor 2008a, 2009). *Pantopsalis*, *Forsteropsalis*, *Neopantopsalis* and *Mangatangi* differ from all *Megalopsalis* species except *Megalopsalis caeruleomontium* by the presence of setae on the mobile finger of the chelicera (Taylor 2013: figs 1d, 2c). *Tercentenarium* has males with a distinct frontodistal bulge on the chelicerae (Taylor 2008b: fig. 3), and females with a ‘keyhole’-like emargination at the front of the genital operculum (Taylor 2008b: fig. 10).

#### Description.

Ozopores usually large, oblong (small, round in *Megalopsalis nigricans*). Dorsum of opisthosoma unarmed (except with transverse rows of spines in *Megalopsalis atrocidiana*). Chelicera segment II denticulate or not; mobile finger usually closing tightly against finger of segment II, fingers bowed apart in larger males of *Megalopsalis caeruleomontium*. Pedipalp usually with patella shorter than tibia (slightly longer in *Megalopsalis nigricans*); apophysis present or absent on patella; claw with ventral tooth-row. Glans relatively short, broad, more or less subtriangular in ventral view, proximal section usually somewhat inflated dorsally (except in *Megalopsalis nigricans*); distal section more or less dorsoventrally flattened. Spiracle with reticulate or partially reticulate covering spines (reduced or absent in *Megalopsalis minima*-species group); lace tubercles present or absent.

#### Distribution

**Figure 1.**
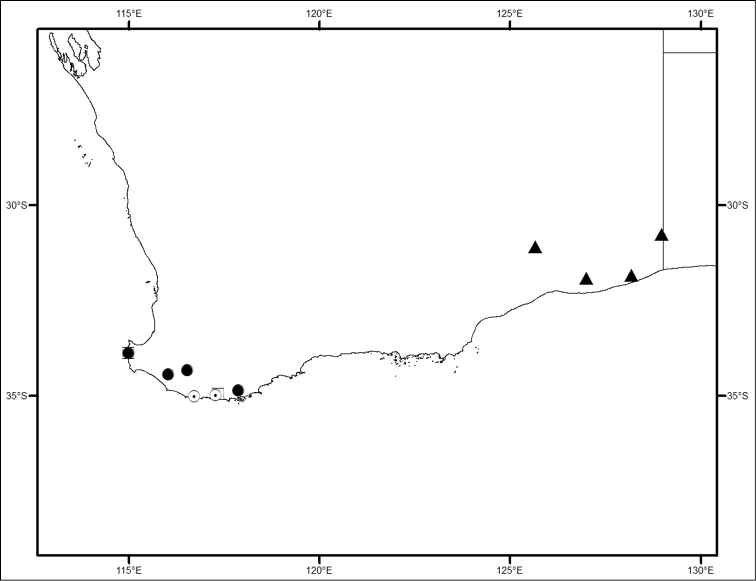
Locality map for *Megalopsalis minima* species-group in southern Western Australia: open square = *Megalopsalis minima*; solid circle = *Megalopsalis porongorupensis*; circle with dot = *Megalopsalis walpolensis*; solid triangle = *Megalopsalis suffugiens*.

(Figs 1, 7). Southern and eastern Australia.

#### Comments.

The genus *Spinicrus* as previously defined (Forster 1949) is likely to be non-monophyletic with regard to both *Megalopsalis* and *Hypomegalopsalis*, and lacks clear synapomorphies (see phylogenetic analysis below). In contrast, the clade uniting these three genera is characterised by a distinct penis morphology, and they are hence united into a single genus *Megalopsalis*. The species groups listed above are clades that were consistently recovered in the phylogenetic analysis under varying analytical conditions; those species not placed in groups did not form consistent subgeneric clades across all analyses. Members of the *Megalopsalis serritarsus*- and *Megalopsalis leptekes*-groups were described by Taylor (2011).

*Megalopsalis tasmanica* and *Megalopsalis thryptica* were described in detail by Hickman (1957), and so are not redescribed here. Both sexes of *Megalopsalis tasmanica* can be distinguished from other Neopilionidae by their distinctive elongate opisthosoma as illustrated by Hickman (1957: fig. 29); this distinction is even more pronounced in the female. See below under *Megalopsalis stewarti* for discussion of the distinction between this species and *Megalopsalis thryptica*.

#### Key to males of species of *Megalopsalis*

**Table:** 

1	Patella of pedipalp with elongate prodistal apophysis	2
–	Patella of pedipalp without apophysis	8
2	Distitarsi III and IV inflated proximally, with ventral rows of brush-like bristles	3
–	Distitarsi III and IV not inflated proximally, without ventral brush-like bristles	7
3	Distitarsus II with ventral swellings on pseudosegments	4
–	Distitarsus II with pseudosegments cylindrical	*Megalopsalis hoggi*
4	Femur II with ventral spines	5
–	Femur II unarmed	*Megalopsalis pilliga*
5	Pedipalpal femur with dorsal spines	*Megalopsalis epizephyros*
–	Pedipalpal femur unarmed or with ventral spines only	6
6	Spiracle spines relatively robust, lace tubercles short and forming more extensive field; pedipalpal femur never spinose (New South Wales)	*Megalopsalis serritarsus*
–	Spiracle spines more slender, lace tubercles more elongate but less extensive; pedipalpal femur may have ventral spines (Victoria, South Australia)	*Megalopsalis eremiotis*
7	Pedipalpal femur with dorsal and ventral spine rows	*Megalopsalis leptekes*
–	Pedipalpal femur unarmed	*Megalopsalis tanisphyros*
8	Dorsum of opisthosoma with transverse rows of spines raised on mounds	*Megalopsalis atrocidiana*
–	Dorsum of opisthosoma unarmed	9
9	Ozopore openings very small, circular, not raised on lateral lobes; pedipalpal femur relatively long, more than 1.5× length of prosoma	*Megalopsalis nigricans*
–	Ozopore openings oblong, raised on lateral lobes; pedipalpal femur shorter than or subequal to prosoma	10
10	Mobile finger of chelicera with ring of setae near central tooth	*Megalopsalis caeruleomontium*
–	Mobile finger of chelicera without setae	11
11	Distitarsi III and IV with ventral rows of brush-like bristles	12
–	Distitarsi III and IV without ventral brush-like bristles	15
12	Opisthosoma distinctly elongate, about 1.5× as long as wide	*Megalopsalis tasmanica*
–	Opisthosoma not elongate, not much longer than wide	13
13	Dorsum of prosoma entirely unarmed	*Megalopsalis sublucens*
–	Dorsum of prosoma strongly denticulate	14
14	Distitarsus IV inflated proximally	*Megalopsalis thryptica*
–	Distitarsus IV evenly cylindrical	*Megalopsalis stewarti*
15	Segment II of chelicera denticulate; spiracle with covering spines absent (Western Australia)	16
–	Segment II of chelicera unarmed; covering spines present over spiracle (eastern Australia)	19
16	Dorsum of prosoma strongly denticulate	*Megalopsalis minima*
–	Dorsum of prosoma unarmed or with very few denticles	17
17	Legs unarmed or with sparse, relatively long and slender spines; body silvery	*Megalopsalis suffugiens*
–	Legs with numerous small denticles; opisthosoma with dark transverse stripes	18
18	Pedipalp with numerous denticles on femur and patella	*Megalopsalis porongorupensis*
–	Pedipalp unarmed	*Megalopsalis walpolensis*
19	Ocularium spinose	*Megalopsalis coronata*
–	Ocularium unarmed	*Megalopsalis puerilis*

### Megalopsalis minima

(Kauri, 1954) comb. n.

http://species-id.net/wiki/Megalopsalis_minima

Fig. 3

Spinicrus minimus Kauri, 1954: 7–8, fig. 4a–b (incorrect original spelling).
Spinicrus minimum Kauri – Taylor 2004: 76 (spelling emended therein by W. Staręga).

#### Material examined.

1 minor male, Denmark, Western Australia, 34°57'S, 117°21'E, 11 November 1990, A. F. Longbottom, under granite (WAM T72865); 3 minor males, Glenbourne farm, Old Ellensbrook Rd, S of Gracetown, Western Australia, 33°53'S, 115°00'E, 27–28 October 1996, L. Marsh et al., pitfall (WAM T72171, T72184 [2 measured]); 3 major males, ditto, 28–30 June 1997, L. Marsh et al., dry pitfalls, base of cliff (WAM T72167–9; measured); 2 minor males, ditto, 13–15 September 1997, L. Marsh et al., dry pitfall traps (WAM T72176 [measured], T72186); 1 minor male, ditto, 27–29 December 1997, L. Marsh et al., dry pitfalls, site 3 (WAM T72160); 1 minor male, 33°54'28"S, 115°00'49"E, 24–26 October 1998, L. Marsh et al., dry pitfall traps (WAM T72172; measured); 2 minor males, ditto, 33°54'32"S, 115°00'24"E, 24–26 October 1998, L. Marsh et al., dry pitfall traps (WAM T72158); 2 minor males, ditto, 33°54'40"S, 115°00'34"E, 30 October–1 November 1999, L. Marsh et al., dry pitfall traps (WAM T72144; 1 measured); 1 minor male, ditto, 20–22 October 2001, L. Marsh et al., dry pitfall traps (WAM T72193; measured); 1 minor male, ditto, 33°55'08"S, 115°00'44"E, 20–23 October 2000, L. Marsh et al., dry pitfall traps (WAM T72187; measured).

#### Diagnosis.

*Megalopsalis minima* can be distinguished from other members of the *Megalopsalis minima*-species group by the heavier denticulation on the dorsal prosomal plate (Fig. 3a); the major males can also be distinguished from other species by the proportionately much longer chelicerae (Fig. 3b). It can be distinguished from *Megalopsalis porongorupensis* by the absence of spines on the pedipalpal femur and patella, and from *Megalopsalis suffugiens* by the heavily denticulate leg femora (Fig. 3b).

**Figure 2.**
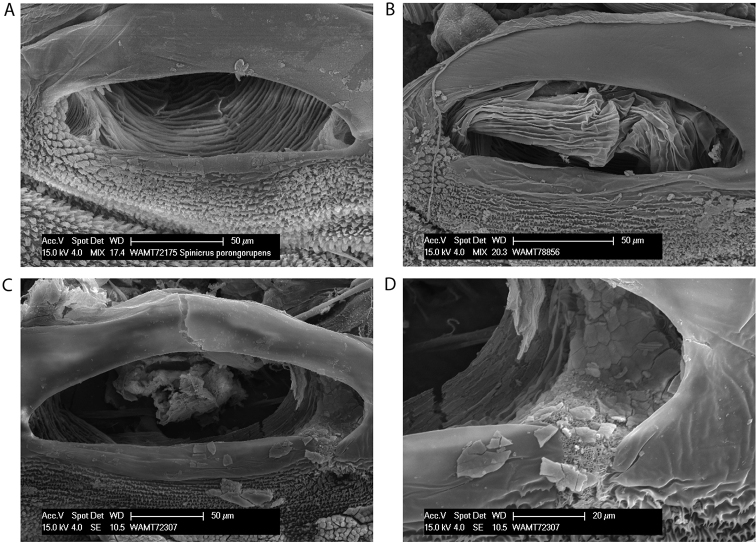
Spiracles of *Megalopsalis minima* species-group: **a** *Megalopsalis porongorupensis* **b** *Megalopsalis walpolensis* **c** *Megalopsalis suffugiens* **d** same, close-up of lateral corner showing area of reticulation.

**Figure 3.**
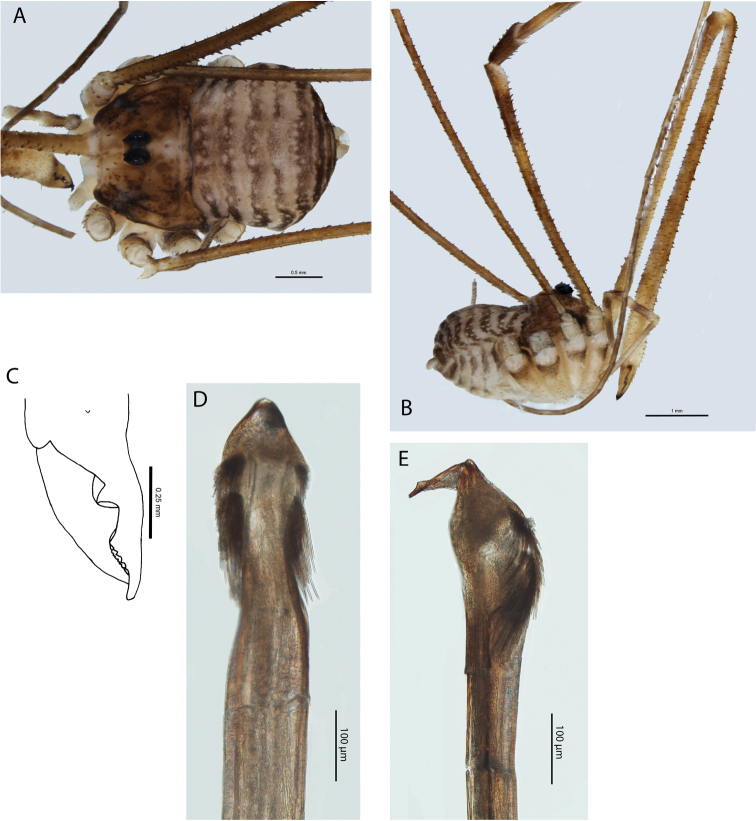
*Megalopsalis minima*, major male (all WAM T72169): **a** body, dorsal view **b** body, lateral view **c** right cheliceral fingers, frontal view **d** glans, ventral view **e** glans, right lateral view.

#### Description.

MAJOR MALE (N = 3). Prosoma length 0.85 (0.78–0.90), width 1.86 (1.74–1.92); total body length 2.37 (2.18–2.56). Dorsal prosomal plate golden brown; median prosomal area strongly denticulate, fewer denticles on margins of anterior and posterior prosomal areas. Ocularium black with row of denticles along edge on each side. Ozopore large, lenticulate. Dorsum of opisthosoma with alternating tan and dark brown mottled with tan stripes, and scattered iridescent white patches. Coxae tan with medium brown distal ends; venter of opisthosoma dark brown medially; tan dusted with black laterally.

*Chelicerae*. Segment I 5.81 (4.78–7.00), segment II 6.83 (6.10–7.88). Chelicerae golden brown with second segment tan distad; evenly denticulate. Fingers long; mobile finger crescent-shaped (Fig. 3c).

*Pedipalps*. Femur 0.96 (0.89–1.00), patella 0.44 (0.43–0.46), tibia 0.55 (0.54–0.59), tarsus 1.23 (1.21–1.26). Alternating tan and brown bands; femur without denticles. Femur to proximal part of tibia with longitudinal rows of large setae, distal tibia and tarsus with large setae interspersed among small setae. Inner dorsal distal patella with swelling but no distinct apophysis. Microtrichia on distal end of tibia and tarsus; claw with ventral tooth-row.

*Legs*. Femora 4.27 (3.82–4.55), 7.51 (6.92–7.92), 3.72 (3.48–3.96), 5.75 (5.33–5.94); patellae 0.87 (0.80–0.98), 0.96 (0.92–1.07), 0.81 (0.76–0.86), 0.95 (0.93–1.00); tibiae 3.96 (3.62–4.28), 8.15 (7.42–8.46), 3.67 (3.44–3.84), 5.79 (5.27–5.98). Femora with strong denticles. Patella I with two longitudinal rows of spines, one on each side; rows continue on tibia, dwindling distalwards. Patellae of other legs only lightly denticulate; tibiae smooth. Tibia II with 7–9 pseudosegments, tibia IV with two pseudosegments.

*Penis* (Figs 3d–e). Tendon long; waist in shaft behind bristle groups; left anterior bristle group reduced. Glans short, triangular in ventral view, not strongly flattened distally; dorsal side in line with shaft, evenly convex. Deep pores.

*Spiracle*. Spines almost entirely absent, residual reticulate bases only towards lateral corner; dense field of lace tubercles at lateral corner.

MINOR MALE (N = 7). Prosoma length 0.73 (0.55–0.83), width 1.82 (1.39–1.68); total body length 1.83 (1.45–2.25). As above, except for following.

*Chelicerae*. Segment I 0.96 (0.66–2.61), segment II 1.68 (1.27–3.44).

*Pedipalps*. Femur 0.84 (0.78–0.90), patella 0.37 (0.34–0.39), tibia 0.48 (0.43–0.53), tarsus 1.05 (0.93–1.10).

*Legs*. Femora 3.48 (3.20–3.70), 6.57 (6.27–6.79), 3.34 (3.00–3.52), 4.79 (4.30–5.00); patellae 0.72 (0.63–0.80), 0.83 (0.77–0.87), 0.72 (0.69–0.76), 0.83 (0.70–0.85); tibiae 3.46 (2.84–3.80), 7.02 (6.33–7.34), 3.26 (2.72–3.52), 4.74 (4.15–5.00). Patella I lightly denticulate, without longitudinal spine rows.

#### Comments.

Unfortunately, the type specimen(s) of *Megalopsalis minima* were not available for the present study. This species has been identified based on its original description by Kauri (1954).

Females have been found in association with males of *Megalopsalis minima*, *Megalopsalis porongorupensis* and *Megalopsalis walpolensis* (unpublished observations, specimens in WAM). However, as no distinct morphotypes have been distinguished among the likely females, while the ranges of these species overlap, it has not been possible as yet to determine which females are assignable to which species.

### Megalopsalis porongorupensis

(Kauri, 1954) comb. n.

http://species-id.net/wiki/Megalopsalis_porongorupensis

Figs 2a , 4

Spinicrus porongorupensis Kauri, 1954: 8–9, fig. 4c (incorrect original spelling).
Spinicrus porongorupense Kauri – Taylor 2004: 76 (spelling emended therein by W. Staręga).

#### Material examined.

5 males, Glenbourne, Old Ellensbrook Road, S of Gracetown, Western Australia, 33°53'S, 115°00'E, 27–28 October 1996, L. Marsh et al., pitfalls (WAM T72175 [2 measured]; T72184 [1 measured]); 2 males, ditto, 27–29 December 1997, L. Marsh et al., dry pitfalls (WAM T72152–3; measured); 1 male, ditto, 33°54'50"S, 115°00'57"E, 24–26 October 1998, L. Marsh et al., dry pitfall traps (WAM T72161; measured); 1 male, ditto, 33°54'32"S, 115°00'24"E, 20–23 October 2000, L. Marsh et al., dry pitfall traps (WAM T72200); 1 male, ditto, 33°54'40"S, 115°00'34"E, 24-26 October 1998, L. Marsh et al., dry pitfall traps (WAM T72173); 1 male, ditto, 25–27 October 2003, L. Marsh et al., dry pitfall traps (WAM T72198; measured); 1 male, ditto, 33°54'35"S, 115°00'15"E, 30 October–1 November 1999, L. Marsh et al., dry pitfall traps (WAM T72155; measured); 3 males, ditto, 33°54'50"S, 115°00'57"E, 30 October–1 November 1999, L. Marsh et al., dry pitfall traps (WAM T72143; 2 measured); 1 male, Pemberton, Crowea Block, Western Australia, 240 m, 17 December 1976, S. J. Curry, pitfall trap (WAM 90/1319); 2 males, ditto, 24 October 1977, S. J. Curry, ridge site, pitfall traps (WAM 90/1321–2); 1 male, ditto, 31 October 1977, S. J. Curry, ridge site, pitfall trap (WAM 90/1335); 1 male, ditto, 11 November 1977, S. J. Curry, ridge site, pitfall trap (WAM 90/1326); 1 male, Porongurup Range, Western Australia, 20 January 1932, E. W. Bennett (WAM 32/217); 1 male, Porongurup National Park, Porongurups, Western Australia, 34°40'55.8"S, 117°51'58.6"E, 570 m, 13 June 1996, S. Barrett, wet pitfalls (WAM T72214); 3 males, Mordalup Road, Unicup, Western Australia, 34°19'01"S, 116°31'49"E, 15 Oct 1999–31 Oct 2000, P. van Heurck, wet pitfalls (WAM T73035).

#### Diagnosis.

*Megalopsalis porongorupensis* is distinguishable from other members of the *Megalopsalis minima*-species group by the presence of denticulation on the pedipalp (Kauri 1954).

#### Description.

MALE (N = 10). Prosoma length 0.81 (0.65–0.91), width 1.55 (1.41–1.74); total body length 1.94 (1.70–2.33). Dorsal prosomal plate including ocularium tan with dark mottling; unarmed. Ozopore large. Dorsum of opisthosoma tan with iridescent white spots and broad white median stripe.

**Figure 4.**
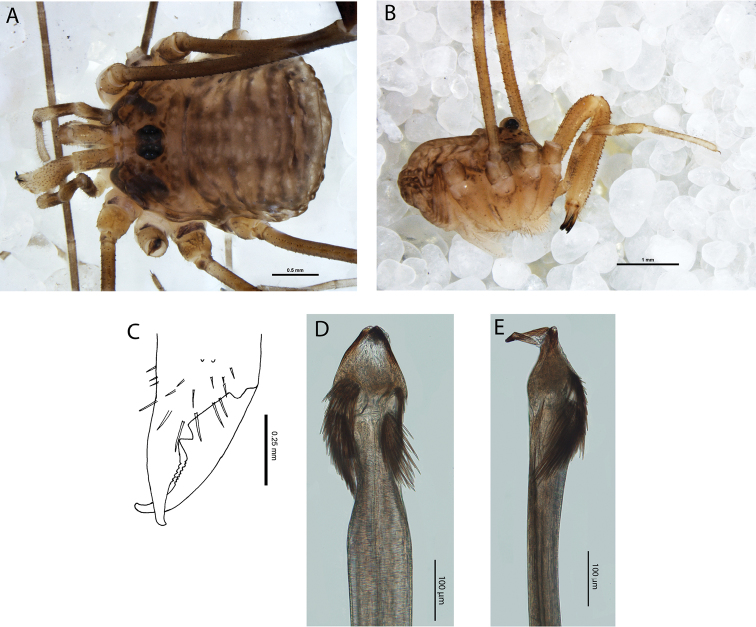
*Megalopsalis porongorupensis*, male: **a** body, dorsal view (WAM T72311) **b** body, lateral view (WAM T72203) **c** left cheliceral fingers, frontal view (WAM T72175) **d** glans, ventral view (WAM T72175) **e** glans, dorsolateral view (WAM T72175).

*Chelicerae*. Segment I 1.35 (0.69–2.20), segment II 2.04 (1.23–3.00). Tan; heavily and uniformly denticulate. Cheliceral fingers medium length; mobile finger crescent-shaped (Fig. 4c).

*Pedipalps*. Femur 0.83 (0.75–1.00), patella 0.38 (0.31–0.45), tibia 0.45 (0.40–0.52), tarsus 1.05 (0.94–1.19). Tan. Femur and patella heavily denticulate, few scattered large setae only; tibia lightly denticulate proximally. Inner dorsal distal patella slightly bulging but no distinct apophysis. Microtrichia on distal part of tibia and tarsus; claw with ventral tooth row.

*Legs*. Femora 3.46 (3.08–3.92), 6.68 (6.00–7.34), 3.24 (2.92–3.48), 5.20 (4.65–5.67); patellae 0.73 (0.66–0.84), 0.84 (0.67–0.89), 0.66 (0.56–0.78), 0.80 (0.64–1.00); tibiae 3.16 (2.88–3.42), 7.12 (6.60–7.83), 3.03 (2.70–3.24), 4.78 (4.15–5.27).

*Penis* (Figs 4d–e). Glans short, dorsal edge in line with shaft; stylus at 90° to glans and shaft. Left anterior bristle group reduced; waist in shaft behind bristle groups. Deep pores.

*Spiracle* (Fig. 2a). Spines almost entirely absent, residual reticulate bases only towards lateral corner; dense field of lace tubercles at lateral corner.

#### Comments.

Unfortunately, the type specimen(s) of *Megalopsalis porongorupensis* were not available for the present study. This species has been identified based on its original description by Kauri (1954), who clearly figured the denticulate pedipalp.

This species shows a relatively large degree of difference in cheliceral size between the largest and smallest individuals, but there is no clear clustering into a larger and a smaller morph.

### Megalopsalis suffugiens sp. n.

http://zoobank.org/EA5093FB-A854-4AE1-B512-5789F226176C

http://species-id.net/wiki/Megalopsalis_suffugiens

Figs 2c–d , 5

#### Material examined.

*Male holotype*. Balgair Station, cave 6N–612, Western Australia, 14 September 1999, N. Poulter, from ceiling adjacent to cave entrance (WAM T72303).

*Paratypes*. 1 male, Balgair Station, cave 6N–1536, Western Australia, 13 September 1999, N. Poulter, walking on damp earth floor (WAM T72299); 1 male, ditto, c. 11 m below cave entrance (WAM T72307); 1 female, Balgair Station, cave 6N-1616, Western Australia, 15 September 1999, P. Devine, N. Poulter, rockhole cave (WAM T72287); 2 males, 1 female, Hampton Tableland, Mundrabilla Station, cave 6N–326, 22 September 1999, P. Devine, N. Poulter, from cave walls in dark zone, largest [female] from entrance lip at night fall (WAM T72298); 1 female, Madura Plains Station (=Moonera Station), cave 6N–1617, 17 September 1999, R. Anderson, N. Poulter, from cave ceiling in dark zone (WAM T72305); 1 female, Nullarbor area, cave 6N–481, 1 October 1994, R. Foulds, from roof of entrance squeeze (WAM T72141).

#### Diagnosis.

**Figure 5.**
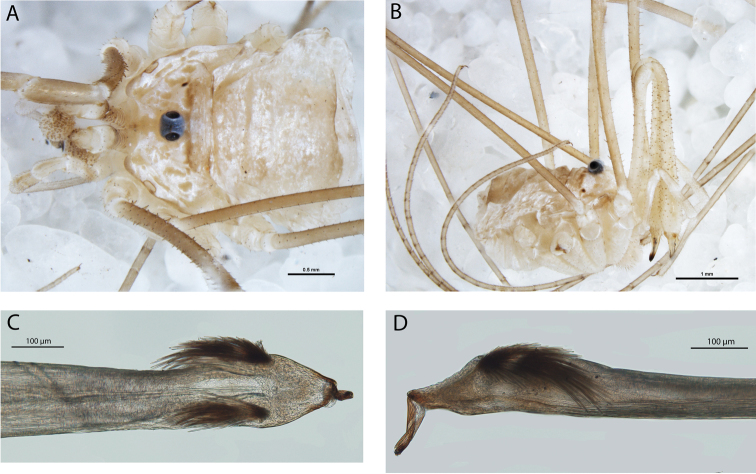
*Megalopsalis suffugiens*, male (all WAM T72299): **a** body, dorsal view **b** body, lateral view **c** glans, ventral view **d** Glans, right lateral view.

*Megalopsalis suffugiens* is readily distinguished from other species of the *Megalopsalis minima*-species group by its pale coloration without dark transverse bands on the opisthosoma (Fig. 5a–b). The spines on the legs (if present) are also proportionately longer and more slender than those found in other species. It can also be distinguished from *Megalopsalis minima* by the lack of denticles on the ocularium and median propeltidial area (Fig. 5a) and from *Megalopsalis porongorupensis* by the lack of denticles on the pedipalps.

#### Description.

MALE (N = 5). Prosoma length 0.75 (0.68–0.83), width 1.69 (1.56–1.86); total body length 2.21 (2.08–2.30). Dorsal prosomal plate unarmed (Balgair Station specimens) or with few denticles on anterior propeltidial area (Hampton Tableland specimens); patched tan and iridescent white with scattered darker mottling. Mesopeltidium with distinct transverse row of black setae. Metapeltidium and anterior part of opisthosoma mottled tan and silver. Posterior part of opisthosoma silver with transverse bands of dark brown mottling.

*Chelicerae*. Segment I 1.47 (0.78–2.22), segment II 2.44 (1.56–3.28). Both segments tan; lightly denticulate with reduced denticulation distad on both segments. Segment II slightly inflated distad. Cheliceral fingers long, slender; mobile finger crescent-shaped.

*Pedipalps*. Femur 1.01 (0.87–1.09), patella 0.51 (0.50–0.54), tibia 0.57 (0.54–0.64), tarsus 1.27 (1.20–1.33). White with tan patches and black setae; femur with longitudinal rows of setae; patella and tibia with black setae laterally and medially, midline glabrous; no apophysis. Microtrichia over greater part of tarsus and tibia; claw with ventral tooth row.

*Legs*. Femora 3.83 (3.17–4.45), 7.14 (6.38–7.77), 3.10 (2.66–3.36), 4.94 (4.10–5.56); patellae 0.90 (0.83–0.99), 1.05 (0.98–1.12), 0.85 (0.78–0.94), 0.99 (0.90–1.06); tibiae 3.71 (3.34–4.00), 7.42 (6.81–7.89), 3.57 (2.90–4.05), 4.91 (4.10–5.52). Trochanters iridescent white; unarmed or with single anterior spine on trochanters I and III. Legs tan; femur I with sparse, slender spines, reduced to only a few dorsally in some specimens; femur II unarmed or with few spines near base; remaining segments unarmed. Femora and patellae with scattered black setae; tibiae and tarsi densely covered in small setae. Tibia II with 11 to 13 pseudosegments; tibia IV with two or three pseudosegments.

*Penis* (Figs 5c–d). Shaft broad, tendon relatively short; bristle groups well-developed. Glans short, broad, triangular in dorsal view; in line with shaft; dorsal side evenly convex; not significantly flattened distally. Pores shallowly recessed.

*Spiracle* (Figs 2c–d). No occluding spines; lace tubercles at lateral corner reduced to patch of reticulation.

FEMALE (N = 4). Prosoma length 1.16 (0.61–1.45), width 2.13 (1.98–2.28); total body length 3.83 (3.40–4.40). As for male, except for following: Dorsum unarmed.

*Chelicerae*. Segment I 0.75 (0.66–0.81), segment II 1.58 (1.55–1.62). Unarmed.

*Pedipalps*. Femur 1.31 (1.28–1.34), patella 0.68 (0.65–0.71), tibia 0.78 (0.73–0.83), tarsus 1.65 (1.63–1.67). Patella and tibia more densely setose medially than male.

*Legs*: Femora 4.68 (4.45–4.95), 9.28 (8.73–10.23), 4.01 (3.72–4.28), 5.95 (5.63–6.16); patellae 1.18 (1.07–1.24), 1.42 (1.32–1.45), 1.11 (1.03–1.19), 1.19 (1.13–1.24); tibiae 4.79 (4.65–4.96), 9.77 (9.12–10.38), 4.44 (4.28–4.63), 6.05 (5.81–6.25). Femora and patellae with longitudinal rows of small spines.

#### Variation.

Males show a noticeable variation in the size of the chelicerae that correlates with the development of armature on the legs; however, the variation is not as large as that found in *Megalopsalis minima*, and it is uncertain at present whether variation is continuous or a distinction occurs between major and minor males. Further specimens are also required to establish whether the difference in dorsal armature recorded above between Balgair Station and Hampton Tableland specimens indicate separate populations in these localities.

#### Etymology.

From the Latin *suffugio*, to take shelter, to reflect the finding of specimens of this species within caves in the arid Nullarbor.

#### Comments.

All specimens of *Megalopsalis suffugiens* recorded to date were collected in caves; however, *Megalopsalis suffugiens* does not show any obvious troglobitic adaptations. The eyes remain well-developed and the legs are proportionately only slightly longer than in other *Megalopsalis* species. It seems more likely that *Megalopsalis suffugiens* only uses the caves as damp refugia during the day, emerging at night to feed. This suggestion is supported by the collection of at least one specimen (WAM T72298) from a cave entrance at nightfall.

### Megalopsalis walpolensis sp. n.

http://zoobank.org/9ACD0DA5-BE0D-4FCB-BEB7-D2E4F195C8E9

http://species-id.net/wiki/Megalopsalis_walpolensis

Figs 2b , 6

#### Material examined.

Male holotype. Walpole-Nornalup National Park, Knoll Drive, Walpole, Western Australia, 34°59'43"S, 116°43'12"E, 29 October 2006, M. L. Moir, A. Sampey (WAM T78848).

*Paratype*. 1 male, Mt Shadforth, Western Australia, 34°58’04"S, 117°16’47"E, 6 November 2006, M. L. Moir, D. Jolly, in leaf litter (WAM T78856).

#### Diagnosis.

**Figure 6.**
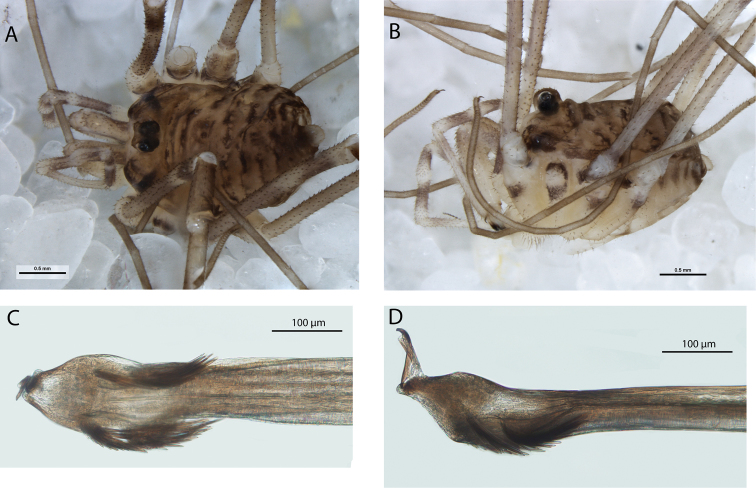
*Megalopsalis walpolensis*, male (all WAM T78848): **a** body, dorsal view **b** body, lateral view **c** glans, ventral view **d** glans, left lateral view.

The features of *Megalopsalis walpolensis* appear intermediate between those of *Megalopsalis minima* and *Megalopsalis porongorupensis*. It differs from *Megalopsalis minima* in lacking significant denticulation on the ocularium and propeltidium (Fig. 6a) and from *Megalopsalis porongorupensis* in lacking denticles on the pedipalps.

**Figure 7.**
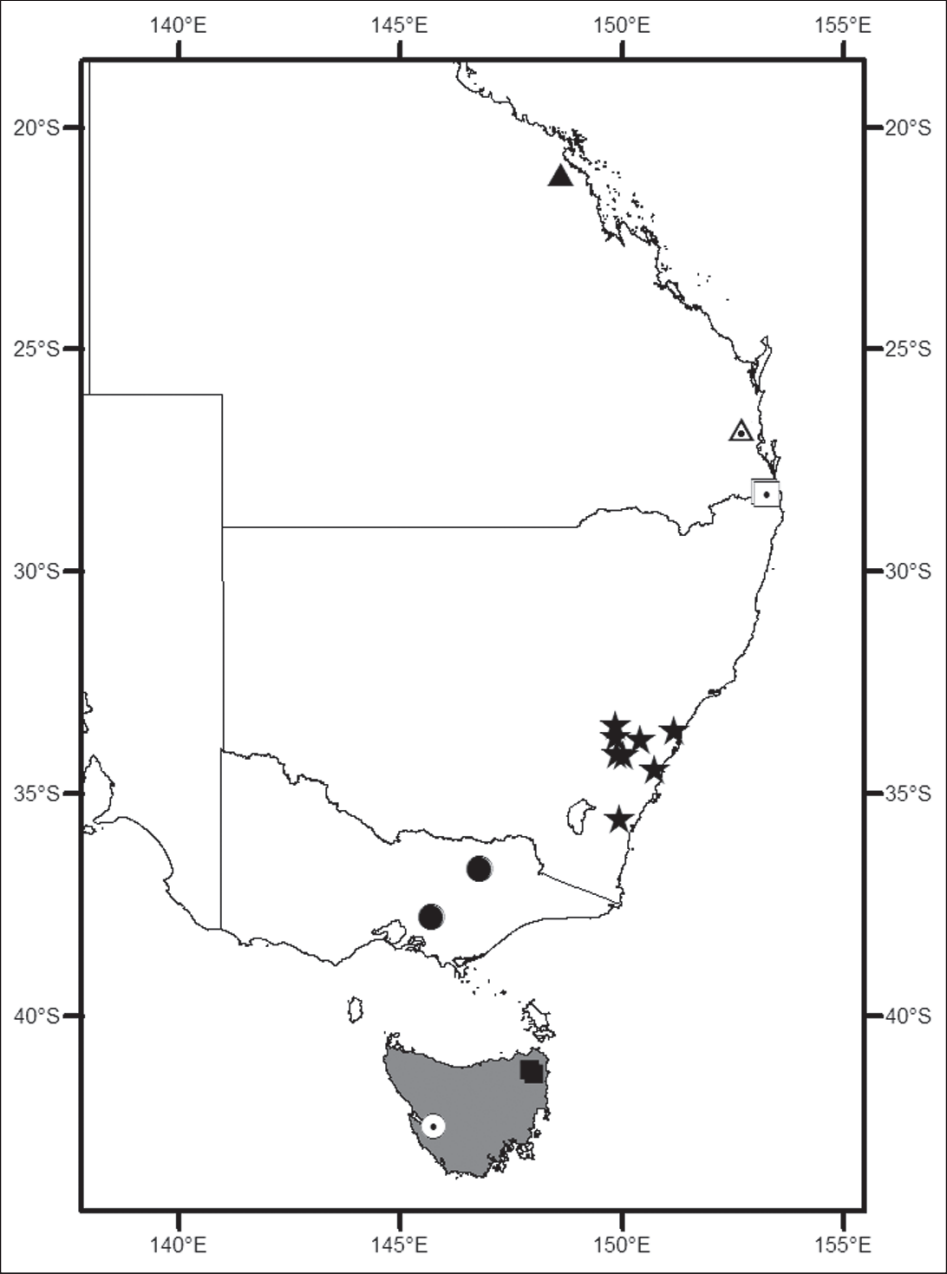
Locality map for *Megalopsalis* species (excluding *Megalopsalis serritarsus*-group) in eastern Australia: grey shading = *Megalopsalis tasmanica* and *Megalopsalis nigricans*; circle with dot = *Megalopsalis sublucens*; solid square = *Megalopsalis thryptica*; solid circle = *Megalopsalis stewarti*; solid star = *Megalopsalis caeruleomontium*; square with dot = *Megalopsalis coronata*, triangle with dot = *Megalopsalis puerilis*, solid triangle = *Megalopsalis atrocidiana*.

#### Description.

MALE (N = 2). Prosoma length 0.65 (0.55–0.74), width 1.44 (1.34–1.53); total body length 2.22 (2.13–2.30). Anterior propeltidial area cream, remainder of propeltidium golden-brown with mottled black patches on anterior corners of dorsal prosomal plate and lateral shelves. Prosoma mostly unarmed, except few small scattered denticles on lateral edge of dorsal prosomal plate near odoriferous glands. Odoriferous glands visible as black patches through cuticle. Ocularium dark golden-brown, with row of small low denticles around each eye. Mesopeltidium, metapeltidium and opisthosoma with transverse band of mottled black across golden-brown background of each segment, broken by tan or iridescent white spots. Coxae cream with mottled purple patches at distal ends; venter of opisthosoma cream dusted with purple, condensing to more solid patches laterally.

*Chelicerae*. Segment I 0.73 (0.67–0.79), segment II 1.42 (1.25–1.59). Segment I mottled purple on cream background with purple mottling more solid laterally than medially; scattered denticles dorsally. Segment II cream, mottled with purple proximally, densely denticulate proximally with denticles thinning until distal third is unarmed. Cheliceral fingers short, lateral margin evenly rounded.

*Pedipalps*. Femur 0.90 (0.89–0.91), patella 0.42 (0.40–0.44), tibia 0.50 (0.48–0.52), tarsus 1.11 (1.07–1.14). Cream banded with purple, with cream stripe down dorsal midline; unarmed. No patellar apophysis; black setae denser on medial side of patella but not hypersetose. Microtrichia on tarsus, except for proximal third, and distalmost end of tibia. Tooth-comb on claw.

*Legs*. Femora 3.48 (3.44–3.52), 6.20 (6.15–6.24), 3.33 (3.32–3.34), 5.05 (4.98–5.11); patellae 0.81 (0.78–0.83), 1.05 (1.03–1.06), 0.82 (0.81–0.83), 0.95; tibiae 3.42 (3.40–3.44), 6.95 (6.93–6.96), 3.16 (3.13–3.19), 4.90 (4.84–4.96). Trochanters white-cream mottled with purple, unarmed. Femora golden-brown proximally, with cream band beginning distad of halfway, followed by purple band, then cream distal end. Patellae dark cream dusted with black, tibiae and metatarsi banded cream and dusty black, tarsi cream. Femora and distal ends of patellae with broken rows of dorsal denticles, remaining segments unarmed. Tibia II with seven pseudosegments, tibia IV undivided.

*Penis* (Figs 6c–d). Left anterior bristle group somewhat reduced, remaining bristle groups well-developed. Glans short, broad, triangular in dorsal view; roughly in line with shaft; dorsal side evenly convex; not significantly flattened distally. Deep pores.

*Spiracle* (Fig. 2b). Spines entirely absent; dense patch of lace tubercles at lateral corner.

#### Etymology.

From the type locality, Walpole, with the suffix –*ensis* indicating geographic origin.

### Megalopsalis atrocidiana sp. n.

http://zoobank.org/10CF153B-F43C-4FFE-9511-EC6BE7320776

http://species-id.net/wiki/Megalopsalis_atrocidiana

Figs 8 , 9a–c

#### Material examined.

*Male holotype*. Central Queensland, Mt Dalrymple, 21°03'S, 148°38'E, 1200 m, 21 December 1992–10 January 1993, ANZSES Expedition, flight intercept trap (QM S35935).

*Paratype*. 1 female, ditto (QM S35935).

#### Diagnosis.

**Figure 8.**
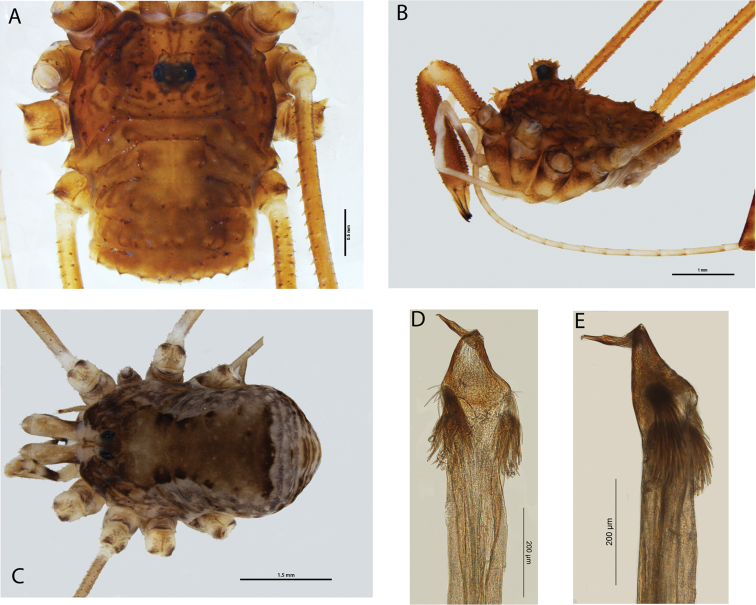
*Megalopsalis atrocidiana* (all QM S35935): **a** body of male, dorsal view **b** body of male, lateral view **c** body of female, dorsal view **d** glans, ventral view **e** glans, right lateral view.

*Megalopsalis atrocidiana* differs from all other long-legged Enantiobuninae in the presence of transverse rows of spines on the opisthosoma (Fig. 8a); these are present in reduced form in the females as well as the males.

#### Description.

MALE (N = 1). Prosomalength 2.18, width 1.26; total body length 2.66. Body medium brown; darker mottling on prosoma. Dorsal prosomal plate sharply denticulate; denticles along posterior margins of prosomal segments. Lateral spines on each side of metapeltidium. Ocularium with high spines. Ozopore large. Opisthosoma with transverse rows of spines on raised mounds along midlines of first four segments. Coxae golden brown with dark brown patches distally; venter of opisthosoma light grey-brown.

*Chelicerae*. Segment I 1.40, segment II 2.53. Segment II darker than segment I; distal end of segment I white. Both segments evenly denticulate. Cheliceral fingers long, mobile finger angular crescent-shaped.

**Figure 9.**
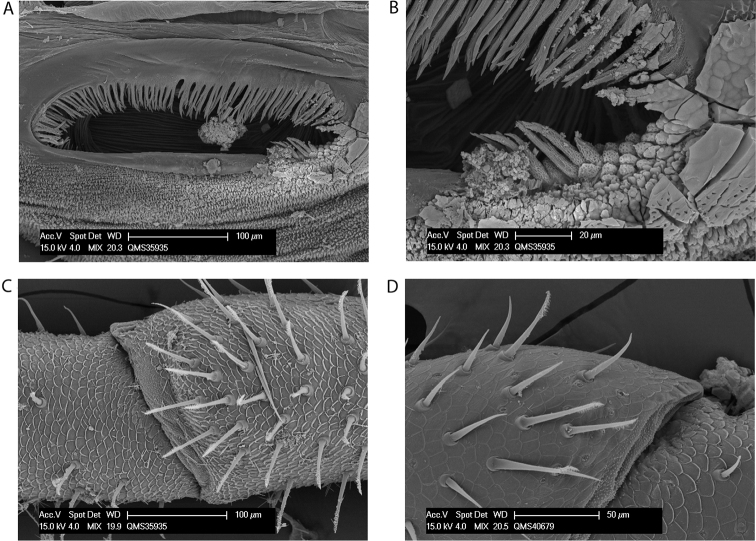
*Megalopsalis atrocidiana* and *Megalopsalis coronata*, SEM images: **a** *Megalopsalis atrocidiana*, spiracle **b** same, close-up of lateral corner **c** *Megalopsalis atrocidiana*, right pedipalp, medial view of patella and tibia, showing plumose setae **d** *Megalopsalis coronata*, left pedipalp, medial view of distal end of patella, showing mixture of plumose and non-plumose setae.

*Pedipalps*. Femur 1.15, patella 0.53, tibia 0.63, tarsus 1.42. Proximal half of femur brown, distal half of femur to tibia white, tarsus tan. Unarmed; no apophysis on patella. Plumose setae present medially (Fig. 9c). Microtrichia on distal three-quarters of tarsus; claw with ventral tooth-row.

*Legs*. Femora –, 6.77, 3.48, 5.41; patellae –, 1.19, 1.01, 1.11; tibiae –, 7.89, 3.24, 5.03. Golden brown. Trochanters with robust spines on prolateral face. Leg I not preserved. Femora of remaining legs denticulate; patellae with longitudinal rows of small denticles; remaining segments unarmed. Tibia II with seven or eight pseudosegments; tibia IV undivided.

*Penis* (Figs 8d–e). Left anterior bristle group slightly reduced, remaining bristle groups well developed. Glans of medium length, edges converging in ventral view.

*Spiracle* (Figs 9a–b). Dense curtain of robust reticulate spines extending partway across spiracle; terminations of spines multifurcate but not palmate; lace tubercles in lateral corner, with small number of reticulate spines at lateral end of posterior margin.

FEMALE (Fig. 8c; N = 1). Prosoma length 1.5, width 2.3; total body length 3.48. Anterior propeltidial area tan, remainder of propeltidium mottled medium brown. Ocularium with row of denticles on each side. Mesopeltidium medially medium brown, laterally tan with black mottling; small denticles medially. Metapeltidium and first three segments of opisthosoma medially medium brown with black patches on edge of medial area, laterally tan mottled with black. Metapeltidium and first four segments of opisthosoma with transverse rows of small denticles. Posterior part of opisthosoma tan mottled with black. Coxae patched tan and dark brown; venter of opisthosoma grey with longitudinal rows of dark brown patches.

*Chelicerae*. Segment I 0.77, segment II 1.65. Segment I tan with dark brown lateral patches proximodorsally; segment II golden brown with tan fingers. Unarmed.

*Pedipalps*. Femur 1.28; patella 0.59; tibia 0.72; tarsus 1.60. Femur dark brown on proximal half, tan on distal half with golden brown patch on distalmost end; patella and tibia each golden brown proximally, tan distally; tarsus tan. Unarmed; no apophysis on patella.

*Legs*: Femora 3.56, 6.77, 3.40, –; patellae 1.17, 1.23, 1.11, –; tibiae 3.84, 7.69, 3.24, –. Banded tan and medium brown; longitudinal dorsal rows of denticles on femora and patellae. Tibia II with eight pseudosegments; leg IV not preserved.

#### Etymology.

From the Latin *atrox*, cruel, and the goddess Diana. The transverse rows of mounds on the opisthosoma are reminiscent of the figure known as Diana of Ephesus, while the epithet ‘cruel’ refers to the addition of a spine on each of the mounds.

### Megalopsalis caeruleomontium sp. n.

http://zoobank.org/F531E2F7-3799-4970-AA4A-26F53A9F95B3

http://species-id.net/wiki/Megalopsalis_caeruleomontium

Figs 10 –11

#### Material examined.

*Male holotype*. Mt Kembla, Sydney Catchment Authority Reserve, New South Wales, 34°26'33"S, 150°44'24"E, 11–15 December 1998, L. Gibson, pitfall traps (AMS KS63019; measured).

*Paratypes*. 2 males, 1 female, Blue Mountains road to Ingar picnic area, New South Wales, 33°46'03"S, 150°24'30"E, 3 October 1996, pitfall trap (AMS KS57166, KS57168; all measured); 3 males, Clyde Mountain, 35°33'S, 149°57'E, 24 October 1968, G. B. M[illedge] (AMS KS65018; measured); 3 males, 2 females, Kirkconnell, 28 May 1972, G. S. Hunt (AMS KS21480; 2 females measured); 1 male, Mt Shivering (near pluviometer), E of Oberon, New South Wales, 23 September 1972, G. S. Hunt (AMS KS21484; measured); 4 males, 6 females, Mt Werong (near pluviometer), New South Wales, 3 July 1972, G. S. Hunt (AMS KS23117; 2 males, 5 females measured); 4 males, 3 females, Muogamarra Nature Reserve, Pacific Highway, 0.7 km SE of Bird Gully Swamp, New South Wales, 33°33'42"S, 151°11'15"E, 2-16 December 1999, M. Gray, G. Milledge, H. Smith, pitfall traps (AMS KS62256; 2 females measured); 1 male, hill NE of Oberon, 10 June 1972, G. S. Hunt (AMS KS21483; measured); 1 male, “Scalloway” pool, Geringong, New South Wales, 23 November 1986, G. Wishart, found ‘walking on water’ (AMS KS17413, measured).

#### Diagnosis.

**Figure 10.**
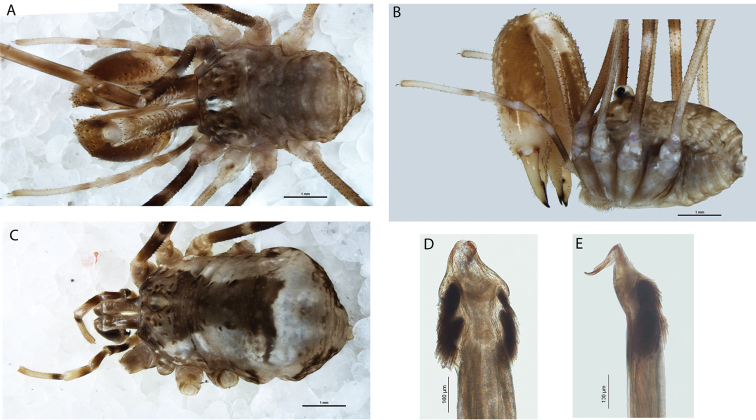
*Megalopsalis caeruleomontium*: **a** body of male, dorsal view (AMS KS63019) **b** body of male, lateral view (AMS KS63019) **c** body of female, dorsal view (AMS KS57168) **d** glans, ventral view (AMS KS63019) **e** glans, right lateral view (AMS KS63019).

*Megalopsalis caeruleomontium* differs from other species of *Megalopsalis* in the presence of setae on the mobile finger of the chelicerae (Fig. 10b). Most males (except the smallest) can also be distinguished by the inflated second segment of the chelicerae (Fig. 10b). *Megalopsalis caeruleomontium* has a relatively flattened penis compared to other *Megalopsalis* species except *Megalopsalis nigricans*; the glans is rather short, with the sides becoming subparallel distally (Figs 10d–e).

#### Description.

MALE (N = 10). Prosoma length 1.17 (0.88–1.46), width 2.34 (1.98–2.50); total body length 3.25 (2.81–3.88). Propeltidium light orange-brown spotted with white and dark brown patches. Anterior propeltidial area pinkish-brown, with diverging dark-brown lines from ocularium to anterior margin, and dark-brown area around short supracheliceral groove on sharply downturned face. Prosoma unarmed. Ocularium bright white with light orange-brown base and behind eyes; postocularium bright white. Mesopeltidium, metapeltidium and first four segments of opisthosoma grey-brown with slightly lighter median band and distinctive transverse row of black setae in lighter spots across each segment. Posterior part of opisthosoma yellow-brown dusted with dark-brown; anal operculum silver. Coxae pinkish-brown with median white areas proximally; venter of opisthosoma grey-brown.

*Chelicerae*. Segment I 2.26 (0.57–3.26), segment II 3.36 (1.24–4.55). Segment I medium-brown dorsally and on proximal two-thirds laterally, peach-coloured ventrally and distolaterally, white patch at distolateralmost end with ventrolateral medium-brown patch directly underneath it; denticulate dorsally and on ventrolateral and ventromedial edges. Segment II strongly inflated in larger specimens to not inflated in smallest specimens, proximally mottled medium-brown and pink dorsally, medium-brown laterally, peach ventrally; distally pink-cream, fingers yellow-cream; denticulate dorsally and ventrolaterally. Cheliceral fingers bowed apart proximally in larger specimens, less or not bowed in smaller specimens.

*Pedipalps*. Femur 1.29 (0–1.46), patella 0.55 (0.37–0.64), tibia 0.78 (0.48–0.95), tarsus 1.63 (1.08–1.91). Trochanter and proximalmost part of femur cream; proximal two-thirds of femur medium-brown, then peach band, then light-brown band; patella pink-brown; tibia pink-brown proximally, cream distally; tarsus pink-brown at proximalmost end, remainder cream. No patellar apophysis. Microtrichia on tarsus and distal third of tibia; tooth-comb on claw.

*Legs*. Femora 4.26 (3.72–4.65), 7.29 (6.50–8.15), 3.97 (3.60–4.25), 5.90 (5.31–6.28); patellae 1.11 (0.91–1.23), 1.26 (1.06–1.44), 1.08 (0.80–1.30), 1.23 (0.97–1.37); tibiae 3.96 (3.31–4.28), 7.35 (6.50–8.00), 3.81 (3.14–4.10), 5.48 (4.67–6.00). Trochanters pinkish-cream, unarmed. Legs I and III medium-brown with cream bands, legs II and IV yellow-brown. Femora denticulate, with larger denticles dorsally than ventrally; fewer denticles dorsally on patellae, remaining segments unarmed.

*Penis* (Fig. 10d–e). Tendon long; bristle groups well-developed. Glans in line with shaft; dorsoventrally flattened for entire length with bases of bristle groups (especially left) consequently more ventral than lateral; glans short, sides converging in ventral view. Deep pores.

**Figure 11.**
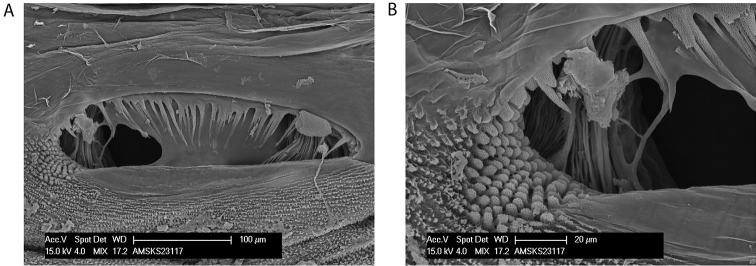
*Megalopsalis caeruleomontium*: **a** spiracle **b** same, close-up of lateral corner.

*Spiracle* (Fig. 11). Sparse curtain of slender reticulate spines extending only partway across spiracle; terminations of spines multifurcate; dense patch of lace tubercles at lateral corner.

FEMALE (Fig. 10c; N = 10). Prosoma length 1.30 (1.03–1.74), width 2.29 (2.08–2.59); total body length 4.60 (3.88–6.13). Propeltidium medium-grey-brown with dark-brown patches; prosoma unarmed. Ocularium grey-brown, unarmed. Mesopeltidium, metapeltidium and first three segments of opisthosoma medially dark-grey-brown, laterally whitish-grey with dark-brown patches on lateral margins. Posterior part of opisthosoma whitish-grey with mottled dark-brown patches laterally. Coxae light-brown mottled with white proximally followed by central cream band, medium-brown distally darkening to dark-brown pro- and retrolaterally. Mouthparts and genital operculum light tan. Venter of opisthosoma medium-orange-brown densely mottled with silver-white.

*Chelicerae*. Segment I 0.84 (0.72–0.95), segment II 1.66 (1.49–1.79). Dark orange-brown reticulated with silver dorsally and large silver-white patch distolaterally on first segment; unarmed.

*Pedipalps*. Femur 1.21 (1.14–1.38), patella 0.55 (0.50–0.59), tibia 0.78 (0.70–0.84), tarsus 1.55 (1.42–1.67). Femur light tan at proximalmost end, remainder medium brown; patella medium brown with small silver patches distolaterally; tibia and tarsus each proximally medium brown, distally light tan silvered dorsally. No apophysis or hypersetose areas; microtrichia over entire length of tibia and tarsus.

*Legs*. Femora 3.57 (3.38–3.92), 6.30 (5.97–7.23), 3.37 (3.17–3.86), 5.20 (4.94–5.63); patellae 1.10 (1.00–1.15), 1.22 (1.15–1.31), 1.06 (0.95–1.15), 1.18 (1.03–1.26); tibiae 3.40 (3.22–3.76), 6.31 (6.07–6.85), 3.26 (3.06–3.60), 4.84 (4.45–5.06). Trochanters grey-tan mottled with white, unarmed. Legs banded medium brown and light tan, with tan bands overlain by silver from distalmost end of femur to tibia. Femora and patellae with longitudinal rows of small denticles.

#### Etymology.

From the Latin words *caeruleus*, blue, and *mons*, mountain: “of the Blue Mountains”, in reference to the distribution of this species.

#### Comments.

Males of this species vary significantly between the largest and smallest individuals in the development of the chelicerae, from inflated with bowed fingers in the largest specimens to slender with unbowed fingers in the smallest. However, variation appears to be more or less continuous (albeit with large individuals distinctly more numerous than small individuals) without a clear division between morphs.

### Megalopsalis coronata sp. n.

http://zoobank.org/ACE42F7E-1AB9-48CE-A102-39E2530A452F

http://species-id.net/wiki/Megalopsalis_coronata

**Figure 12.**
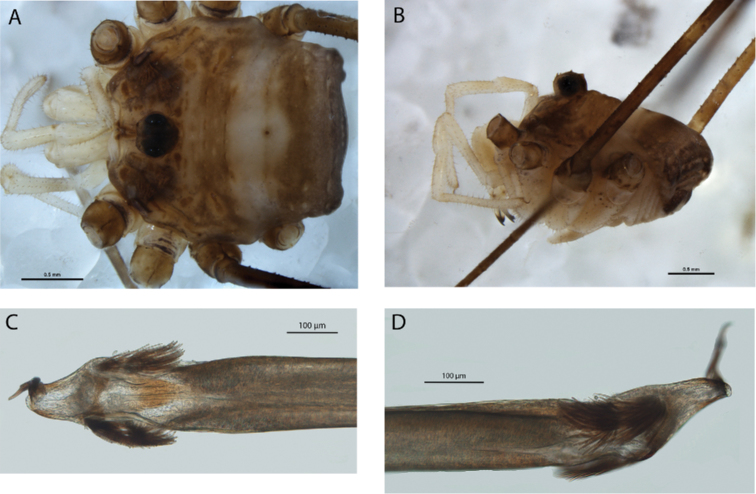
*Megalopsalis coronata*, male (all QM S74237): **a** body, dorsal view **b** body, lateral view **c** glans, ventral view **d** glans, right lateral view.

**Figure 13.**
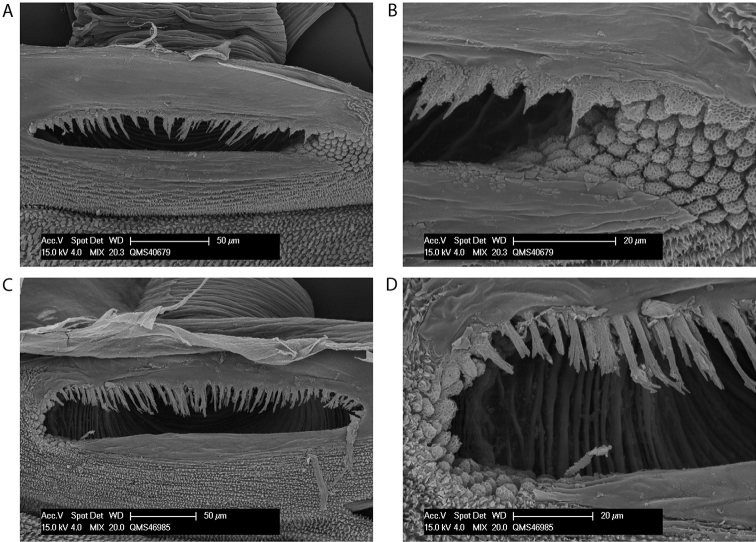
Spiracles of *Megalopsalis* species: **a** *Megalopsalis coronata*, spiracle **b** same, close-up of lateral corner **c** *Megalopsalis puerilis*, spiracle **d** same, close-up of lateral corner.

Figs 9d , 12 , 13a–b

#### Material examined.

*Male holotype*. Queensland, Sunday Creek, 18 December 1996–20 January 1997, G. Monteith, rainforest intercept (QM S40679).

*Paratypes*. 1 male, ditto (QM S40679); 1 male, ditto, Conondale Range, 900 m, 2 March–12 April 1992, D. J. Cook, rainforest pitfall (QM S74237).

#### Diagnosis.

*Megalopsalis coronata* is distinguished from all other *Megalopsalis* species except *Megalopsalis tanisphyros*, *Megalopsalis puerilis* and *Megalopsalis sublucens* by its small, unarmed chelicerae. It is distinguished from *Megalopsalis tanisphyros* by the absence of a pedipalpal patellar apophysis, from *Megalopsalis puerilis* by the presence of denticles on the ocularium, and from *Megalopsalis sublucens* by the absence of ventral brush-like bristles on distitarsi III and IV. The glans of *Megalopsalis coronata* is relatively long compared to other *Megalopsalis* species, with a lower angle of convergence between the sides in ventral view (Fig. 12c).

#### Description.

MALE (N = 3). Prosoma length 0.97 (0.93–1.04), width 1.79 (1.74–1.86); total body length 1.94 (1.78–2.13). Propeltidium golden-brown reticulated with iridescent white, anterior propeltidial area mottled with black; prosoma unarmed. Lateral shelves mostly iridescent white. Mesopeltidium and metapeltidium medially light golden-brown with transverse rows of iridescent white spots, laterally iridescent gold-white. First three segments of opisthosoma medially yellow-brown with iridescent white spots, median area broadening posteriorly; laterally solid gold-white, fading posteriorly, with medium brown edges medially and along boundary of segments I and II. Posterior part of opisthosoma patched white and mottled purple. Coxae I-III yellow-cream; coxae IV and venter of opisthosoma orange.

*Chelicerae*. Segment I 0.48 (0.36–0.57), segment II 1.09 (1.06–1.11). Iridescent white articular membranes between prosoma and chelicerae. Chelicerae white-cream reticulated with iridescent white; unarmed. Fingers long, closing tightly against each other.

*Pedipalps*. Femur 0.89 (0.89–0.90), patella 0.44 (0.42–0.45), tibia 0.53 (0.51–0.55), tarsus 1.09 (1.05–1.12). White-cream, unarmed. Patella with angular mediodistal bulge, but no apophysis; medial side not hypersetose. Small number of plumose setae on mediodistal end of patella only (Fig. 9d). Microtrichia along most of tarsus; claw with ventral tooth row.

*Legs*. Femora 3.60, 6.70 (6.46–6.93), 3.48 (3.36–3.70), 5.45 (5.13–5.94); patellae 0.88, 0.97 (0.95–0.98), 0.80 (0.75–0.85), 0.93 (0.88–0.95); tibiae 3.36, 7.40 (7.38–7.42), 3.06 (2.84–3.36), 4.91 (4.63–5.25). Trochanters medium-brown, unarmed. Legs banded medium-brown and golden-brown; legs I and III predominantly medium-brown, legs II and IV predominantly golden-brown. Femora with scattered denticles, remaining segments unarmed.

*Penis* (Figs 12c–d). Tendon relatively short; bristle groups well-developed. Glans of medium length; sides in ventral view subparallel, converging only slightly; dorsal side angled only slightly dorsad from shaft. Pores with surrounding rim, not raised.

*Spiracle* (Figs 13a–b). Sparse curtain of reticulate spines extending partway across spiracle; spines basally much broader than terminally; terminations of spines simple, pointed, some larger central spines multifurcate; dense patch of lace tubercles at lateral corner.

#### Variation.

The paratype specimens differ in coloration from the holotype, but this may be due to preservation. QM S74237 has the prosoma golden-brown mottled with orange-brown patches, while both QM S74237 and the paratype QM S40679 have a brown transverse band, cream in the former and iridescent white in the latter, across the anterior part of the opisthosoma.

#### Etymology.

From the Latin *coronatus*, crowned, referring to the denticulate ocularium.

#### Comments.

Leg I was only preserved in the holotype.

### Megalopsalis nigricans

(Hickman, 1957) comb. n.

http://species-id.net/wiki/Megalopsalis_nigricans

Fig. 14

Spinicrus nigricans Hickman, 1957: 77, figs 34–40.

#### Material examined.

1 major male, Tasmania, Mount Wellington, 4 January 1969, J. L. Hickman, under logs (AMS KS23719); 1 major male, Tasmania, V. V. Hickman (AMS KS23737); 1 female, SW Tasmania, 12 February 1977, C. Howard, C. Johnson (AMS KS24747); 1 minor male, ditto (AMS KS24749); 2 minor males, NE Tasmania, August 1993 (QM C3.2, C5.1); 1 minor male, 1 female, SW Tasmania, Mount Rufus track, 42°07'S, 146°07'E, 29 April 1987, R. Raven, T. Churchill, open forest, pyrethrum knockdown (QM S1707).

#### Description.

**Figure 14.**
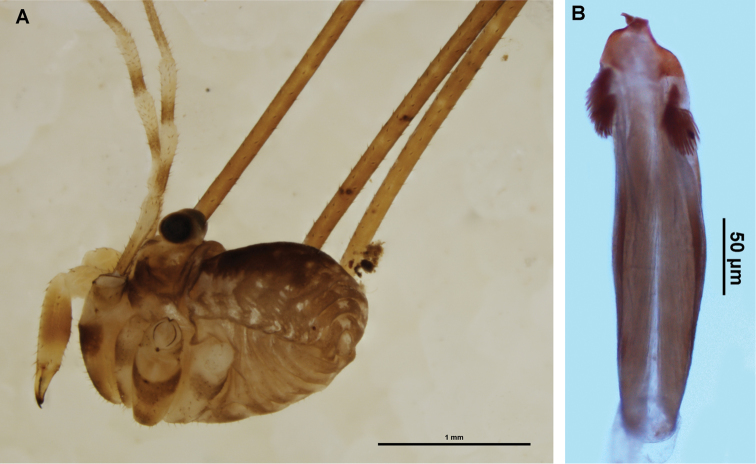
*Megalopsalis nigricans*: **a** minor male, lateral view (AMS KS24749) **b** penis, ventral view (AMS KS23719).

MALE. As for Hickman (1957), except minor male (Fig. 14a): As for major male, except chelicerae not enlarged relative to female. Armature of chelicerae reduced: segment I mostly unarmed with ventral spur, segment II with dorsal longitudinal row of denticles only, with long black seta on each denticle.

*Spiracle* (Taylor 2011: fig. 25). Curtain of partially reticulate spines (reticulation reduced basally) extending partway across spiracle; spines broad, terminations palmate; dense patch of lace tubercles at lateral corner, outer lace tubercles terminally anastomosing.

#### Comments.

This species has already been described in detail by Hickman (1957). As noted above in the discussion of the phylogenetic analysis, this is the most divergent species here assigned to *Megalopsalis*. Its genital morphology is unique: the shaft of the penis is distinctly short and broad (Fig. 14b), in contrast to the elongate shaft of other *Megalopsalis* species (see figures in Taylor 2011). The glans is strongly flattened, not proximally inflated as in other species, and the overall shape is less distinctly subtriangular than in other *Megalopsalis* species, being rather more oblong over the greater part. The ozopores of *Megalopsalis nigricans* are small and round, and not raised on lateral lobes like the larger ozopores of other Enantiobuninae.

Despite Hickman (1957) describing only the major male of this species, collections of this species in AMS and QM indicate that minor males are more abundant and majors relatively uncommon.

### Megalopsalis puerilis sp. n.

http://zoobank.org/6ED54D80-108C-4AFC-BB79-E9B5B92CB5E4

http://species-id.net/wiki/Megalopsalis_puerilis

Figs 13c–d , 15

#### Material examined.

*Male holotype*. SE Queensland, Binna Burra, 27 March 1976, R. Raven, VED, night collection (QM S2835).

*Paratype*. 1 male, Springbrook Repeater, SE Queensland, 1000 m, 28°15'S, 153°16'E, 9 January–19 February 1995, G. B. Monteith, intercept traps (QM S46985).

#### Diagnosis.

**Figure 15.**
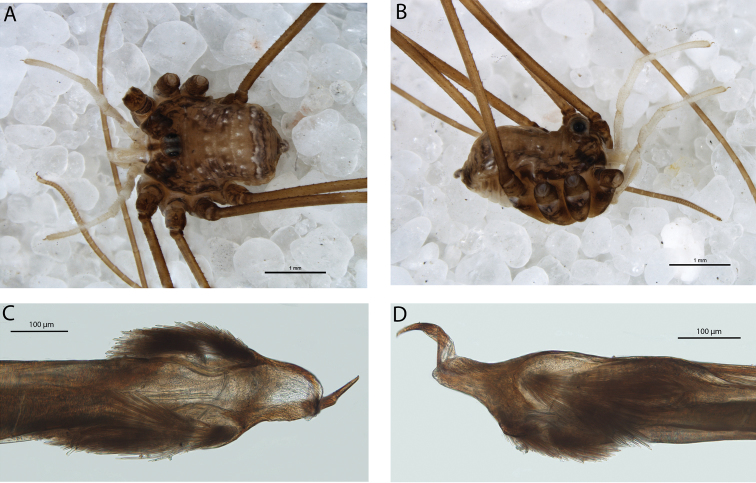
*Megalopsalis puerilis*, male (all QM S2835): **a** body, dorsal view **b** body, lateral view **c** glans, ventral view **d** glans, left ventrolateral view.

*Megalopsalis puerilis* is distinguished from all other *Megalopsalis* species except *Megalopsalis tanisphyros*, *Megalopsalis coronata* and *Megalopsalis sublucens* by its small, unarmed chelicerae. It is distinguished from *Megalopsalis tanisphyros* by the absence of a pedipalpal patellar apophysis, from *Megalopsalis coronata* by the absence of denticles on the ocularium, and from *Megalopsalis sublucens* by the absence of ventral brush-like bristles on distitarsi III and IV. The glans of *Megalopsalis puerilis* is less triangular in overall shape than most other *Megalopsalis* species, with the sides distally subparallel in ventral view (Fig. 15c).

#### Description.

MALE (N = 2). Prosoma length 0.89 (0.83–0.94), width 1.72 (1.56–1.88); total body length 2.37 (2.35–2.38). Dorsum entirely unarmed. Anterior propeltidial area with central stripe of light orange-brown between ocularium and anterior margin of prosoma, flanked by two yellow stripes; remainder of anterior and median propeltidial areas mottled black and dark orange-brown with broad iridescent dark silver patches between ocularium and ozopores. Ocularium iridescent white with dark grey stripe down central groove. Mesopeltidium, metapeltidium and first four segments of opisthosoma orange-yellow medially and along segment boundaries with blackish brown patches laterally; fifth opisthosomal segment with transverse iridescent white stripe bordered by mottled black; remaining segments mottled black with yellow segment boundaries. Mouthparts brown-cream. Coxae dull orange proximally, mottled black distally; venter of opisthosoma iridescent white.

*Chelicerae*. Segment I 0.53 (0.52–0.54), segment II 1.07 (1.00–1.14). Cream; segment I with iridescent white reticulation dorsally. Both segments unarmed. Fingers long; mobile finger closes tightly with segment II.

*Pedipalps*. Femur 0.94 (0.90–0.97), patella 0.45 (0.44–0.45), tibia 0.58 (0.57–0.59), tarsus 1.15 (1.14–1.15). Cream; unarmed. Patella with distomedial bulge, but no true apophysis. Microtrichia on distal three-quarters of tarsus; claw with ventral tooth-comb. *Legs*: Femora 4.16 (3.86–4.45), 7.20 (6.08–8.31), 4.00 (3.76–4.23), 5.65 (5.10–6.19); patellae 0.91 (0.87–0.94), 1.13, 0.87 (0.84–0.89), 1.00 (0.98–1.02); tibiae 4.10 (3.64–4.55), 7.50 (6.46–8.54), 3.74 (3.46–4.02), 5.46 (4.98–5.94). Trochanters mottled black on orange, remaining segments orange-yellow, with widely-spaced mottled black transverse stripes. Femora with scattered denticles, mostly dorsal; other segments unarmed.

*Penis* (Figs 15c–d). Bristle groups well-developed on both sides, left groups set slightly back and longer than right groups. Glans short, sides in ventral view subparallel, dorso-ventrally flattened distally, dorsal surface in plane with shaft. Deep pores.

*Spiracle* (Figs 13c–d). Curtain of robust reticulate spines extending only partway across spiracle; terminations of spines multifurcate but not palmate; lace tubercles on margin of lateral corner only.

#### Etymology.

From the Latin *puerilis*, childish, referring to the lack of ornamentation or significant secondary sexual characteristics in the adult male.

### Megalopsalis stewarti

(Forster, 1949) comb. n.

http://species-id.net/wiki/Megalopsalis_stewarti

Figs 16 , 18a

Spinicrus stewarti Forster, 1949: 68–70, figs 11–16.

#### Material examined.

*Paratypes*. 1 female, Victoria, Mount Buffalo, 30–31 December 1947, ex bole of snow gum (*Eucalyptus pauciflora*) (NMV K-8910); 8 males, ditto (NMV K-8911–8919).

*Other material examined*. 1 male, Victoria, Lala Falls, near Warburton, 37°46'S, 145°42'E, 22 December 2002, M. S. Harvey, M. E. Blosfelds, under bark of *Eucalyptus regnans* (WAM T72315).

#### Description.

MALE. Description as in Forster (1949), except following.

*Legs*. Leg I with anterior longitudinal row of enlarged denticles from proximal end of femur to distal end of tibia (males with smaller chelicerae with reduced denticle row on femur only), scattered denticles on basitarsus I; smaller row on femora and patellae of other legs.

**Figure 16.**
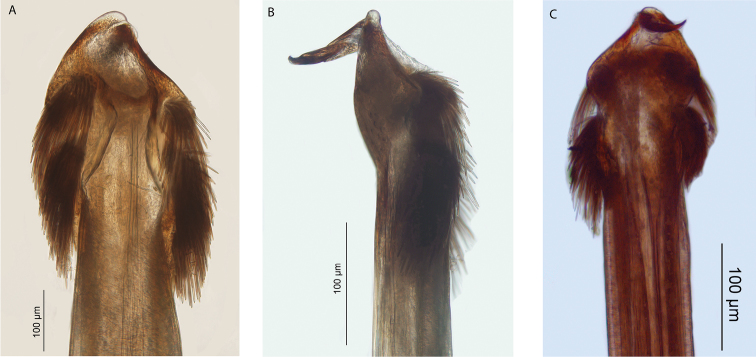
*Megalopsalis stewarti*, male, glans (all WAM T72315): **a** ventral view **b** right lateral view **c** dorsal view.

*Penis* (Fig. 16). Shaft and tendon elongate; all four bristle-groups well-developed. Distinct lateral protrusion of glans above left anterior bristle group. Glans short, broad, triangular in dorsal view, dorsoventrally flattened at distal end. Pores shallowly recessed.

*Spiracle* (Fig. 18a). Reticulate spines extending only partway across spiracle; terminations of spines palmate; small group of lace tubercles in lateral corner.

FEMALE. Description as in Forster (1949), except all legs with dorsal longitudinal row of small denticles along femora.

#### Comments.

Forster (1949) stated that the holotype and female paratype had been deposited in NMV, while the remaining male paratypes (of unspecified number) had been deposited in the Canterbury Museum, Christchurch, New Zealand (CMNZ). However, as indicated above, NMV holds eight male specimens of this species labelled as paratypes. Because these specimens share the same collection data as the female paratype, it seems likely that these correspond to the specimens that Forster (1949) intended to place in CMNZ.

*Megalopsalis thryptica* is very similar to *Megalopsalis stewarti*, from which it can be distinguished by having distitarsus IV basally swollen. Hickman (1957) initially distinguished *Megalopsalis stewarti* from *Megalopsalis thryptica* by the presence of denticles on tibia I in males of *Megalopsalis thryptica*, compared to their supposed absence in *Megalopsalis stewarti* as described by Forster (1949). However, specimens of *Megalopsalis stewarti* with longer chelicerae also have more extensive denticulation on leg I, extending as far as the basitarsus in some specimens. There is insufficient data as yet to determine whether this indicates a division between major and minor males or whether variation is continuous. *Megalopsalis thryptica* may still be distinguished from *Megalopsalis stewarti* by the male of the former having distitarsus IV basally inflated (personal examination of holotype, AMS).

### Megalopsalis sublucens sp. n.

http://zoobank.org/7EAD50EE-61F4-40AB-9EFE-B6164D1596A1

http://species-id.net/wiki/Megalopsalis_sublucens

Figs 17 , 18b–c

#### Material examined.

*Male holotype*. SW Tasmania, Franklin River, below Goodwin’s Peak, January 1983, ANZSS Expedition (QM S2857).

*Paratypes*. 1 male, 1 female, as above (QM S2857).

#### Diagnosis.

**Figure 17.**
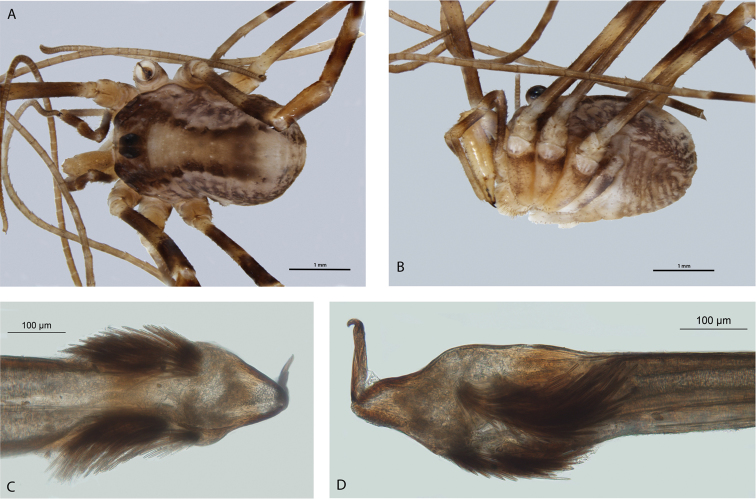
*Megalopsalis sublucens*, male (all QM S2857): **a** body, dorsal view **b** body, lateral view **c** glans, ventral view **d** glans, left ventrolateral view.

*Megalopsalis sublucens* can be readily distinguished from other *Megalopsalis* species by the absence of a pedipalpal patellar apophysis or enlarged chelicerae, in combination with the presence of ventral brush-like bristles on distitarsi III and IV. It can also be distinguished from other species except *Megalopsalis stewarti* by the presence of a lateral protrusion on the left side of the glans near the shaft-glans junction (Fig. 17c).

#### Description.

MALE (N = 2). Prosoma length 1.34 (1.33–1.34), width 1.99 (1.98–2.00); total body length 2.66 (2.59–2.72). Dorsum entirely unarmed. Dark tan stripes from ocularium to anterior margin, remainder of anterior iridescent white. Median propeltidial area white with dark brown patches. Posterior propeltidial area mottled dark brown. Lateral shelves dark brown anteriorly, iridescent white around and posterior to ozopores. Ocularium golden-brown with anterior face silver. Mesopeltidium, metapeltidium and first four segments of opisthosoma medially yellow-brown edged with dark brown with medial iridescent white spots; laterally iridescent white with dark purple mottling. Posterior of opisthosoma white with purple mottling. Venter of prosoma cream, coxae mottled black distally; opisthosoma purple-brown with transverse rows of iridescent white spots.

*Chelicerae*. Segment I 0.68, segment II 1.65 (1.57–1.73). Yellow-cream with tan mottling; not particularly enlarged compared to female. Segment I unarmed, segment II with proximodorsal denticles only. Fingers long, apotele closely opposed to finger of segment II.

*Pedipalp*. Femur 1.13 (1.11–1.15), patella 0.54 (0.50–0.58), tibia 0.70, tarsus 1.35 (1.32–1.38). Banded dark brown and yellow-brown; unarmed; no apophyses. Tooth-comb on apotele.

*Legs*. Femora 3.50 (3.42–3.58), 6.26 (6.13–6.38), 3.28, 5.44 (5.19–5.69); patellae 1.17 (1.13–1.20), 1.27, 1.02, 1.13 (1.06–1.20); tibiae 3.15 (3.07–3.22), 5.48, 3.08, 4.70 (4.50–4.90). Banded dark brown and yellow-brown, with prominent iridescent white spots around accessory spiracles on tibiae. No denticles, but robust spinose setae on all legs to tibiae. Brush-like bristles intermittently present on ventral side of distitarsi III and IV. Femur II not pseudosegmented, tibia II with six pseudosegments, tibia IV with two pseudosegments.

*Penis* (Figs 17c–d). Left anterior bristle group reduced, but left posterior group well-developed. Glans short, triangular in dorsal view, dorsoventrally flattened, dorsal edge in plane with shaft.

**Figure 18.**
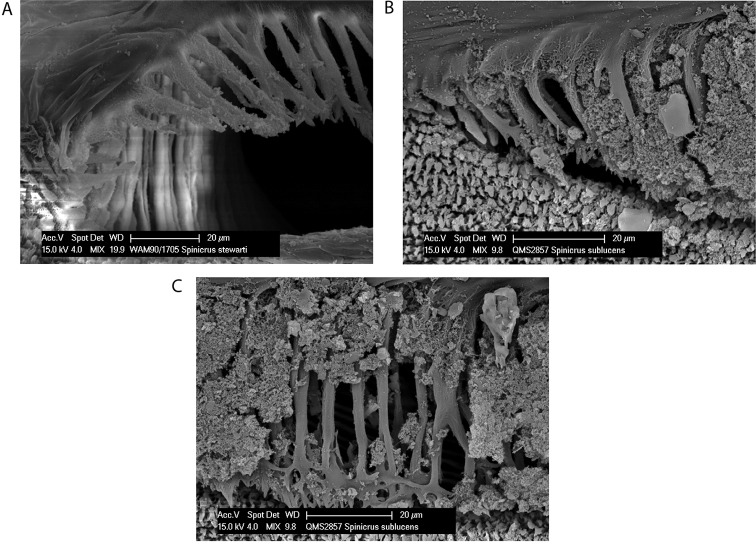
Spiracles of *Megalopsalis* species: **a** *Megalopsalis stewarti*, lateral corner **b** *Megalopsalis sublucens*, lateral corner **c** same, showing anastomosing ends of median spines.

*Spiracle* (Figs 18b–c). Sparse spines, reticulate basally with reticulations fading terminally, extending across spiracle; terminations of spines palmate, anastomosing; no lateral lace tubercles.

FEMALE (N = 1). Prosoma length 1.40, width 2.03; total body length 4.45. As for male except for following. Ocularium iridescent white. Venter of opisthosoma duller.

*Chelicerae*. Segment I 0.75, segment II 1.32. Unarmed.

*Pedipalps*. Femur 1.01, patella 0.50, tibia 0.64, tarsus 1.27.

*Legs*. Femora 2.32, 4.45, 2.28, 3.56; patellae 0.82, 1.01, 0.93, 0.95; tibiae 2.43, 4.30, 2.35, 3.44. Tibia IV undivided.

#### Etymology.

From the Latin *sublucens*, gleaming faintly, in reference to the iridescent patches covering part of the dorsum.

#### Comments.

Those measurements for which a range is not given in the description of the male were only preserved in the holotype.

While *Megalopsalis sublucens* possesses brush-like bristles on distitarsi III and IV as found in *Megalopsalis stewarti*, *Megalopsalis tasmanica* and species of the *Megalopsalis serritarsus*-group, the number of bristles is reduced and they are proportionately more widely spaced and less regular. This may be related to *Megalopsalis sublucens*’ smaller size.

### Phylogenetic analysis

A maximum parsimony analysis was conducted using the programme TNT (Goloboff et al. 2008); apomorphies were mapped onto the resulting trees using the programme Winclada (Nixon 1999). An initial analysis was conducted with all characters given equal weight, followed by successive implied weighting analyses with *k* varying in values of 1.0 from 1.0 to 6.0. Heuristic (“traditional”) searches were conducted using a Wagner-tree random seed, 10 replicates holding 10 trees per replication and constructing trees using a tree bisection-reconnection (TBR) swapping algorithm. Changes from an unambiguous character state to a polymorphic character are counted by TNT as a step, even if the ancestral character state is included in the polymorphism. Jack-knife resampling analysis was conducted with a 36% removal probability over 1000 replications. The character matrix was based on that used in Taylor (2011); see therein for descriptions and discussion of characters not elaborated on below, and for specimen details of outgroup taxa (note that the name *Pantopsalis luna* [Forster, 1944] has been replaced by *Pantopsalis listeri* [White, 1849] as per Taylor 2013). As the monophyly of Eupnoi is well supported (Shultz 1998; Giribet et al. 2002, 2009), representatives of Dyspnoi were excluded in order to reduce the effects of possible homoplasy. The recently described *Mangatangi parvum* Taylor, 2013 was also added to the matrix; see Taylor (2013) for specimen details for this taxon. *Americovibone lanfrancoae* Hunt & Cokendolpher, 1991 was coded based on the description given by Hunt and Cokendolpher (1991). Characters with more than two states have been treated as additive in the order given in initial analyses unless otherwise specified; a further set of analyses was conducted with all characters unordered. The characters used in the analysis are as follows:

1: *Elongate anterior propeltidial area*, *sloping downwards anteriorly*: (0) absent; (1) present.
2: *Ozopore position*: (0) flush with lateral margin of prosoma; (1) raised on protruding lobes.
3: *Ozopore shape*: (0) small and circular; (1) large and oval or oblong.
4: *Raised humps on either side of ocularium*: (0) absent; (1) present.
5: *Raised postocularial hump*: (0) absent; (1) present.
6: *Mesopeltidium*: (0) distinct; (1) merged with propeltidium.
7: *Position of anterior margin of mesopeltidium relative to ocularium*: (0) mesopeltidium immediately behind ocularium; (1) distinct space between ocularium and anterior margin of mesopeltidium.
8: *Metapeltidium*: (0) non-sclerotised; (1) sclerotised.
9: *Dorsal junction between prosoma and opisthosoma*: (0) free; (1) fused.
10: *Dorsum of opisthosoma*: (0) non-sclerotised; (1) sclerotised.
11: *Penis tendon*: (0) long; (1) short.
12: *Angular ventral junction between shaft and glans*: (0) absent; (1) present.
13: *Lateral processes behind shaft-glans articulation*: (0) absent; (1) present.
14: *Bristle groups as lateral processes of penis*: (0) absent; (1) present.
15: *Asymmetry of penis bristle groups*: (0) both sides present; (1) left bristle groups absent in ventral view.
16: *Fused base of lateral bristles on penis*: (0) absent; (1) present.
17: *Dorsal edge of glans*: (0) in same plane as shaft; (1) directed dorsad relative to shaft.
18: *Lateral extension on left side of glans*: (0) absent; (1) present. In *Megalopsalis stewarti* and *Megalopsalis sublucens*, the left side of the glans protrudes outwards above the anterior bristle group (Figs 16c, 17d).
19: *Setae or bristles on glans*: (0) absent; (1) single lateral setae; (2) numerous setae or bristles.
20: *Deeply recessed pores on glans*: (0) absent; (1) present.
21: *Raised pore-bearing papillae on glans*: (0) absent; (1) present. These last two characters were treated as separate states of a single character in Taylor (2011; character 25 therein). This was probably inappropriate: there is no *a priori* reason to believe that a single species could not possess both pore morphologies, and they are here treated separately. Taylor (2011) also differentiated character states for shallowly recessed or shallowly raised genital pores; upon re-examination of the electron micrographs used in coding these states, I am not convinced that they are not artefactual (possibly related to the preservation of the specimen used). I have therefore not included these states in the current analysis.
22: *Glans length*: (0) short; (1) long.
23: *Glans depth*: (0) shallow in lateral view; (1) distinctly deep. In *Pantopsalis albipalpis*, the glans is surmounted by a relatively high, narrow dorsal keel, but the main body of the glans is still recognisable a narrow triangular shape in lateral view, contrasting with the more uniformly deep glans in *Tercentenarium linnaei* (Taylor 2008b) and *Australiscutum* species (Taylor 2009). *Pantopsalis albipalpis* is therefore coded as lacking this character.
24: *Glans shape in ventral view*: (0) subtriangular; (1) parabolic; (2) narrow or constricted. This character replaces character 19 in Taylor (2011), in which the alternate character states were poorly distinguished. A subtriangular glans characterises the species discussed in the current paper (as well as those attributed to *Megalopsalis* in Taylor 2011), in which the sides converge along the greater length of the relatively broad glans. Species of genera such as *Pantopsalis* (Taylor 2004, 2013) and *Australiscutum* (Taylor 2009) have a parabolic glans shape, in which the glans is relatively longer and the rate of convergence of the sides less rapid. Species of outgroup taxa such as Ballarrinae (Hunt and Cokendolpher 1991), as well as *Monoscutum titirangiense* (Taylor 2008a) have a very narrow, elongate glans.
25: *Central concavity on ventral face of glans*: (0) absent; (1) present.
26: *Dorsal side of glans basally inflated*: (0) absent; (1) present (e.g. Figs 3e, 4e).
27: *Shape of distal end of glans*: (0) not dorsoventrally flattened; (1) distinctly dorsoventrally flattened.
28: *Protruding distal end of glans*: (0) absent; (1) present. In species exhibiting this character, the glans is essentially subtriangular in overall shape, but the distal end of the glans has become extended, with the lateral margins becoming more subparallel distally after converging in the proximal part of the glans (Figs 10d, 15c).
29: *Triangular dorsolateral keel on glans*: (0) absent; (1) present.
30: *Sharp dorsal papillae on glans*: (0) absent; (1) present.
31: *Longitudinal membrane on stylus*: (0) absent; (1) present; (2) enlarged. Species of Neopilionidae have a longitudinal membranous banner running along the stylus, and often wrapped around it. This membrane has become particularly large in *Australiscutum*. *Ballarra longipalpus* is coded as lacking this character, though its presence in *Americovibone lanfrancoae* suggests its absence may be secondary for the former species; small membranous flanges at the base of the stylus in *Ballarra* species (Hunt and Cokendolpher 1991) may represent the remnants of a reduced banner.
32: *Number of seminal receptacles*: (0) two; (1) four.
33: *Microsculpture anterior to spiracle*: (0) unornamented; (1) ornamented.
34: *Spiracular entapophysis*: (0) absent; (1) present.
35: *Anterior spines at spiracular aperture*: (0) absent; (1) Dyspnoi-form spines; (2) *Thrasychirus*-form spines or lace tubercles. This character has not been treated as additive. See Taylor (2011) for a discussion of the potential homology or otherwise between the spiracular spines present in Enantiobuninae and those present in Dyspnoi and *Caddo*. *Ballarra longipalpus* was coded by Taylor (2011) as exhibiting a fourth state of this character; however, as illustrated by Taylor (2011: fig. 15), the spiracular spines of *Ballarra longipalpus* are derived from hypertrophy of the micro-ornamentation covering the broader venter, and are almost certainly not directly homologous with the spines present in Enantiobuninae, which are restricted to the spiracle and differ from the surrounding micro-ornamentation. The character state ‘*Ballarra*-form spines’ has therefore been removed from the current analysis, and *Ballarra longipalpus* has been coded as lacking this character. *Neopilio australis*, previously coded as unknown for this character, has been re-coded as possessing *Thrasychirus*-form spines on the basis of the figure provided by Hunt and Cokendolpher (1991).
36: *Reticulate anterior spiracular spines*: (0) absent; (1) present.
37: *Extent of anterior spines over spiracle*: (0) absent; (1) halfway; (2) entire spiracle.
38: *Terminations of anterior spiracular spines*: (0) simple; (1) palmate.
39: *Lace tubercles at corner of spiracle*: (0) absent; (1) present. *Megalopsalis suffugiens* possesses a patch of lace-like reticulation marking the position occupied by the lace tubercles in other taxa, and has been coded as possessing this character.
40: *Posterior margin of spiracle*: (0) unornamented; (1) short ornamentation; (2) elongate spines.
41: *Ventral spur at base of cheliceral segment I*: (0) absent; (1) present.
42: *Ventrolateral row of enlarged denticles on cheliceral segment I*: (0) absent; (1) present.
43: *Cheliceral segment II compared to segment I*: (0) not significantly inflated; (1) inflated.
44: *Cheliceral finger length*: (0) short; (1) long.
45: *Mobile finger of chelicera*: (0) closes tightly against immobile finger of segment II; (1) bows away from immobile finger proximally.
46: *Setae on mobile finger of chelicera*: (0) absent; (1) present.
47: *Medial side of pedipalpal coxae*: (0) unarmed; (1) with covering of blunt denticles.
48: *Plumose setae on pedipalp*: (0) absent; (1) present.
49: *Length of pedipalpal femur*: (0) shorter than or subequal to prosoma length; (1) more than 1.5 × as long as prosoma.
50: *Pedipalpal patella vs tibia*: (0) patella shorter than tibia; (1) patella longer than tibia.
51: *Medial side of pedipalpal patella*: (0) sparsely setose; (1) hypersetose.
52: *Apophysis on pedipalpal patella*: (0) absent; (1) poorly developed (less than one-third of patella length); (2) well-developed (about one-half of patella length). In Taylor (2011), the presence of a pedipalpal patellar apophysis was coded separately for males and females; this risked inappropriately weighting the significance of the patellar apophysis as the two characters are closely correlated. Instead, the presence of a pedipalpal patellar apophysis has here been coded as a single character, with a second character (character 53 below) coded only for species possessing the apophysis to account for the reduction of the apophysis in the male of *Pantopsalis* species relative to the females.
53: *Pedipalpal patellar apophysis in male*: (0) reduced relative to female apophysis; (1) fully developed as in female.
54: *Shape of apophysis on pedipalpal patella*: (0) rounded; (1) triangular. As with character 52, this character has been coded for both males and females rather than the two being coded separately as in Taylor (2011). The only species in which males and females are known to differ in apophysis shape, *Forsteropsalis grimmetti*, has been coded as polymorphic for this character.
55: *Pedipalp with reflexed tibia relative to patella*: (0) absent; (1) present. A dorsally reflexed pedipalpal tibia is characteristic of the Ballarrinae (Hunt and Cokendolpher 1991).
56: *Shape of pedipalpal tibia*: (0) straight; (1) bent mediad from patella.
57: *Distribution of microtrichia on pedipalp*: (0) absent; (1) distal half to third of tarsus only; (2) full length of tarsus; (3) tibia and tarsus.
58: *Pedipalpal claw*: (0) reduced or absent; (1) present. *Neopilio australis* has been coded as possessing a reduced pedipalpal claw, as per Hunt and Cokendolpher (1991).
59: *Teeth on pedipalpal claw*: (0) absent or only one or two teeth; (1) tooth-comb.
60: *Armature of coxa I*: (0) absent; (1) present.
61: *Armature of trochanter I*: (0) absent; (1) present in the form of prolateral denticles only; (2) present in the form of prolateral and retrolateral denticles; (3) present in the form of retrolateral denticles only. This character has not been treated as additive.
62: *Leg I length and shape*: (0) long and slender; (1) short and sturdy.
63: *Leg I armature in male*: (0) absent; (1) present on femur; (2) present on femur to patella; (3) present on femur to tibia; (4) present on femur to basitarsus; (5) present on femur to distitarsus.
64: *Arrangement of denticles on leg I*: (0) scattered; (1) sublinear; (2) linear.
65: *Prolateral longitudinal row of hypertrophied spines on leg I*: (0) absent; (1) present.
66: *Pseudoarticulations in femur II*: (0) absent; (1) present.
67: *Accessory tracheal stigmata in tibiae*: (0) absent; (1) present.
68: *Tibia II shape*: (0) cylindrical; (1) swollen.
69: *Pseudoarticulations in tibia II*: (0) absent; (1) present.
70: *Pseudoarticulations in tibia IV*: (0) absent; (1) present.
71: *Pseudoarticulations in basitarsi*: (0) absent; (1) present.
72: *Ventrodistal spines on basitarsal pseudosegments*: (0) absent; (1) present.
73: *Mobile hinge between basitarsus and distitarsus*: (0) absent; (1) present.
74: *Ventrodistal spines on the junction of the basitarsus and distitarsus*: (0) absent; (1) present.
75: *Ventrodistal swelling on pseudosegments of distitarsus II*: (0) absent; (1) present.
76: *Ventral double row of brush-like bristles on distitarsi III and IV*: (0) absent; (1) present.
77: *Ventrodistal spines on distitarsal pseudosegments*: (0) absent; (1) present.
78: *Proximal part of distitarsi III and IV*: (0) not swollen; (1) swollen.

## Results and discussion

**Figure 19.**
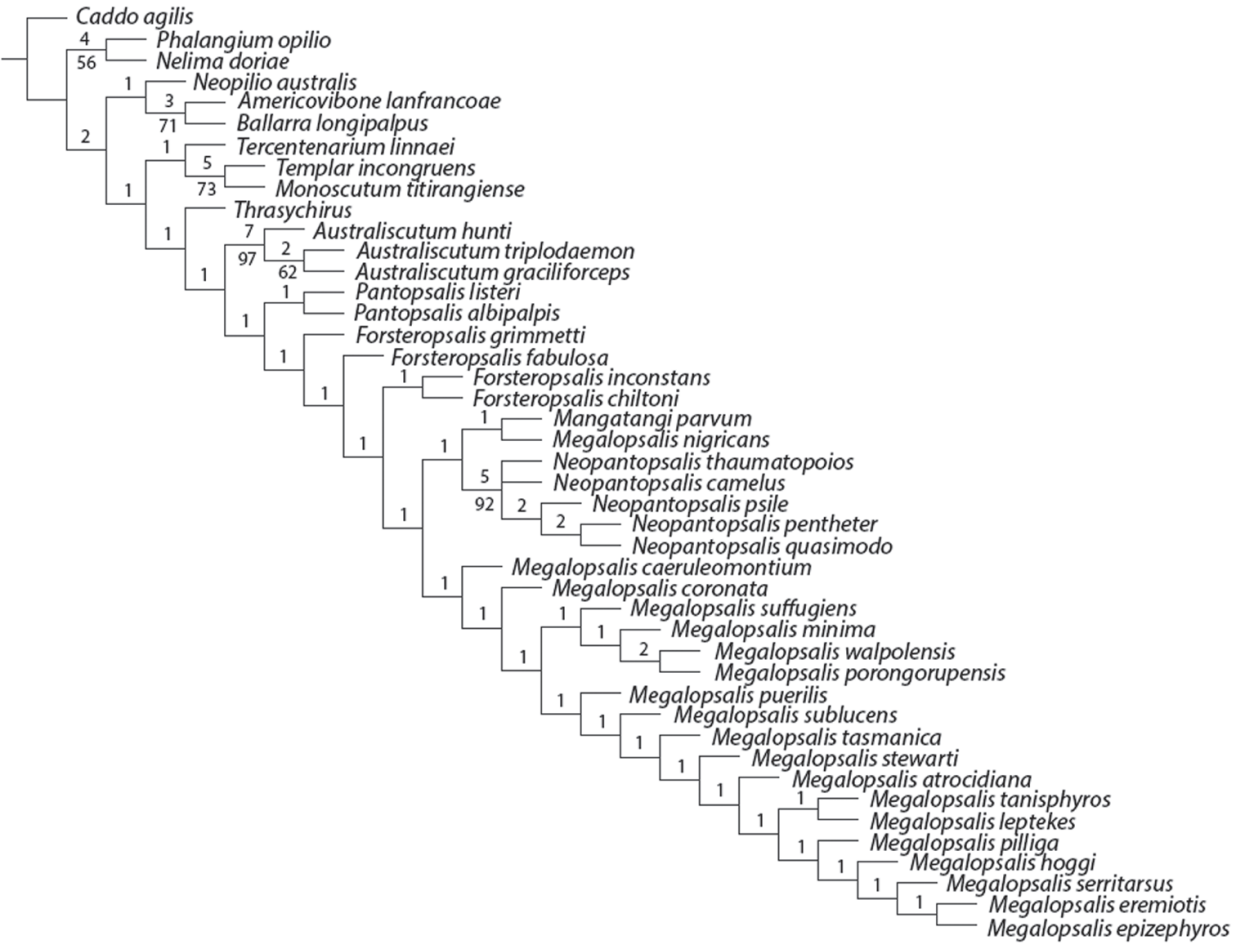
Consensus from equal weights analysis with multistate characters ordered. Numbers above branches indicate Bremer support values; numbers below branches indicate jack-knife support. Those branches without lower numbers received less than 50% jack-knife support.

**Figure 20.**
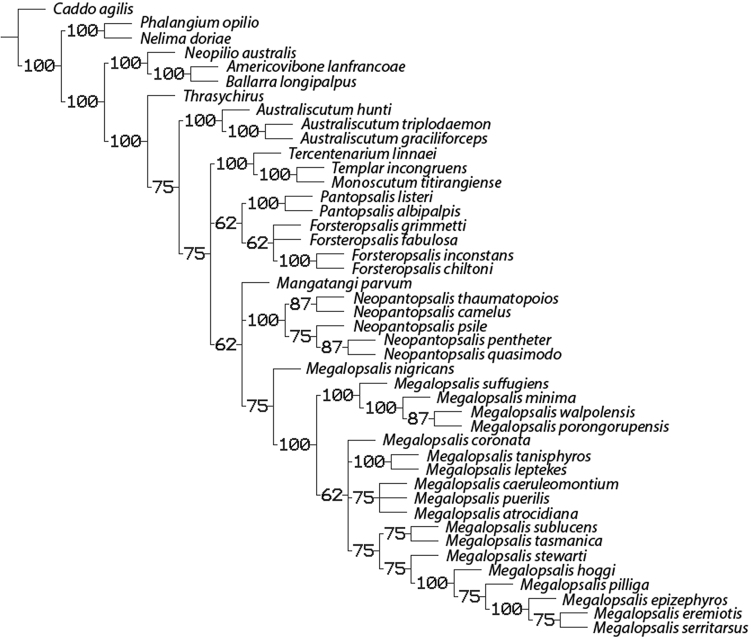
50% majority rule consensus of trees resulting from equal and implied weights analyses with multistate characters ordered. Numbers on branches indicate percentage of trees in which the given clade was recovered.

The analysis with all characters given equal weight, and multistate characters treated as ordered, produced two equally supported trees of 321 steps (CI = 0.290, RI = 0.626), the consensus of which is shown in Fig. 19. Bremer support for most clades was low, and very few can be regarded as numerically well supported. Nevertheless, monophyly was recovered for Neopilionidae (including Ballarrinae) and Enantiobuninae *sensu* Taylor (2011). Enantiobuninae was placed as sister to a clade of *Neopilio australis* and Ballarrinae. All implied weights analyses returned a single best tree, agreeing with the equal weight analysis on the monophyly of Neopilionidae and Enantiobuninae. Figure 20 shows the majority-rule consensus for all trees recovered under various weighting schemes with ordered characters; Table 1 shows support for selected clades under individual weighting schemes.

**Table 1.** Plot of recovery for selected clades under varying weight analyses with ordered characters. Gray fill indicates monophyly; white fill indicates para- or polyphyly. Abbreviations used: *Mang*. = *Mangatangi*; *Pant*. = *Pantopsalis*; *Forst*. = *Forsteropsalis*; *M*. = *Megalopsalis*. See text for definition of *Megalopsalis minima*- and *Megalopsalis serritarsus*-groups.<br/>

*k* value	0.0	1.0	2.0	3.0	4.0	5.0	6.0
Neopilionidae							
Enantiobuninae							
Australasian Enantiobuninae							
*Australiscutum*							
*Mang*. + *Pant*. + *Forst*.							
*Pantopsalis* + *Forsteropsalis*							
*Pantopsalis*							
*Forsteropsalis*							
*Neopantopsalis*							
*Megalopsalis*							
*Megalopsalis* excl. *M. nigricans*							
*M. minima*-group							
*M. serritarsus*-group							

**Figure 21.**
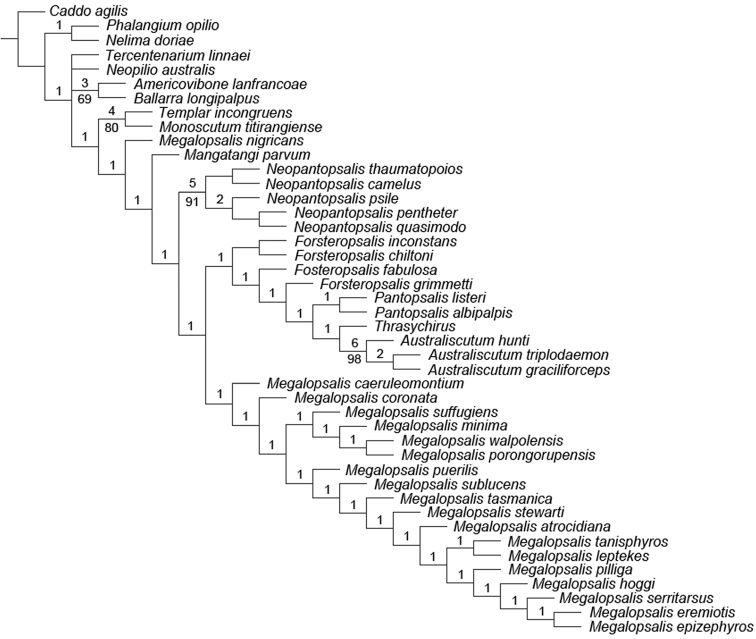
Consensus from equal weights analysis with multistate characters unordered. Numbers above branches indicate Bremer support values; numbers below branches indicate jack-knife support. Those branches without lower numbers received less than 50% jack-knife support.

**Figure 22.**
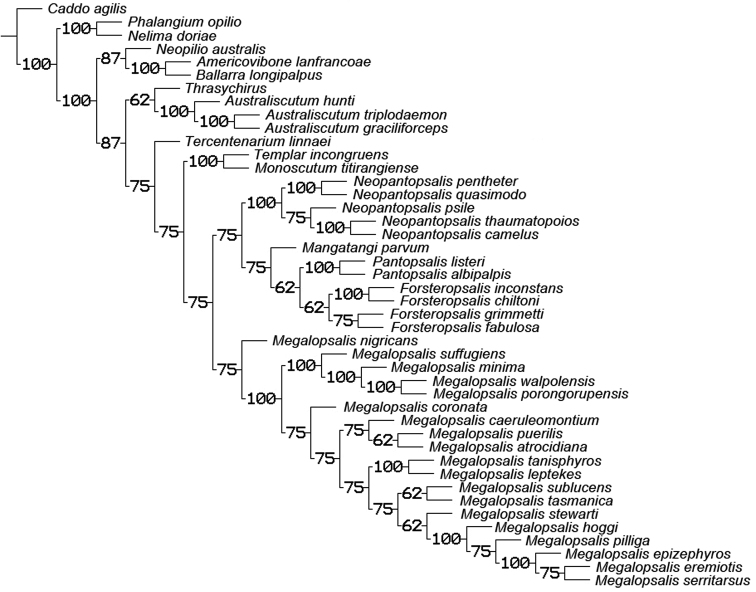
50% majority rule consensus of trees resulting from equal and implied weights analyses with multistate characters unordered. Numbers on branches indicate percentage of trees in which the given clade was recovered.

**Figure 23.**
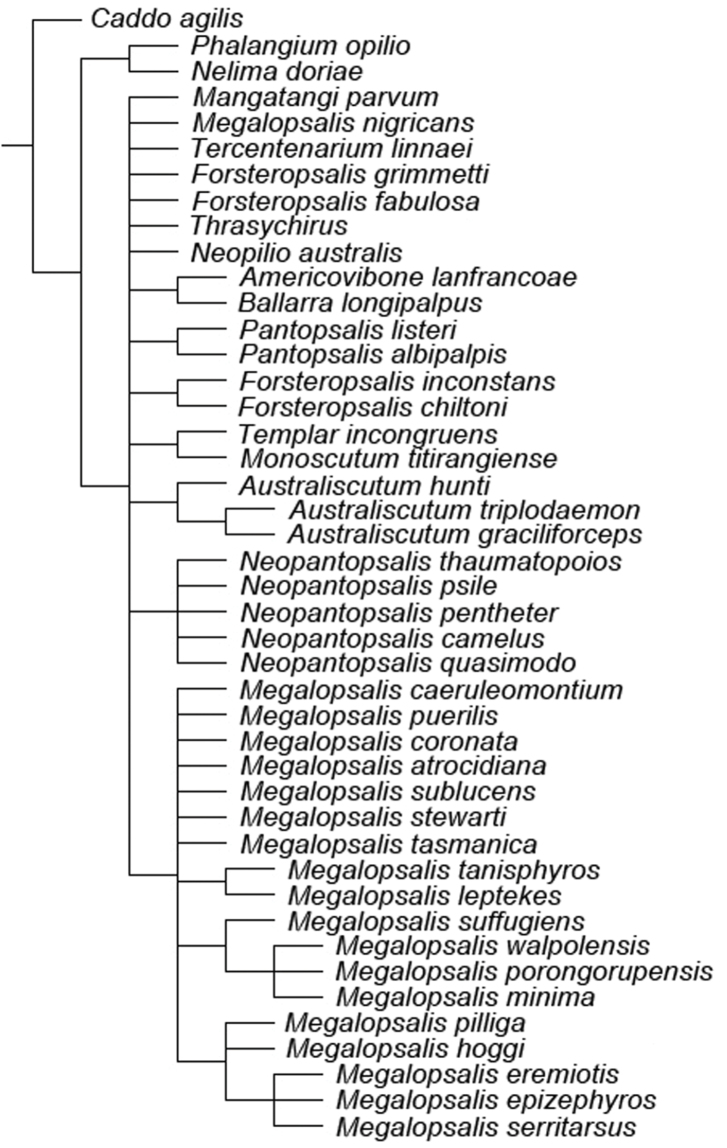
Strict consensus of all trees recovered under all analytical conditions.

**Figure 24.**
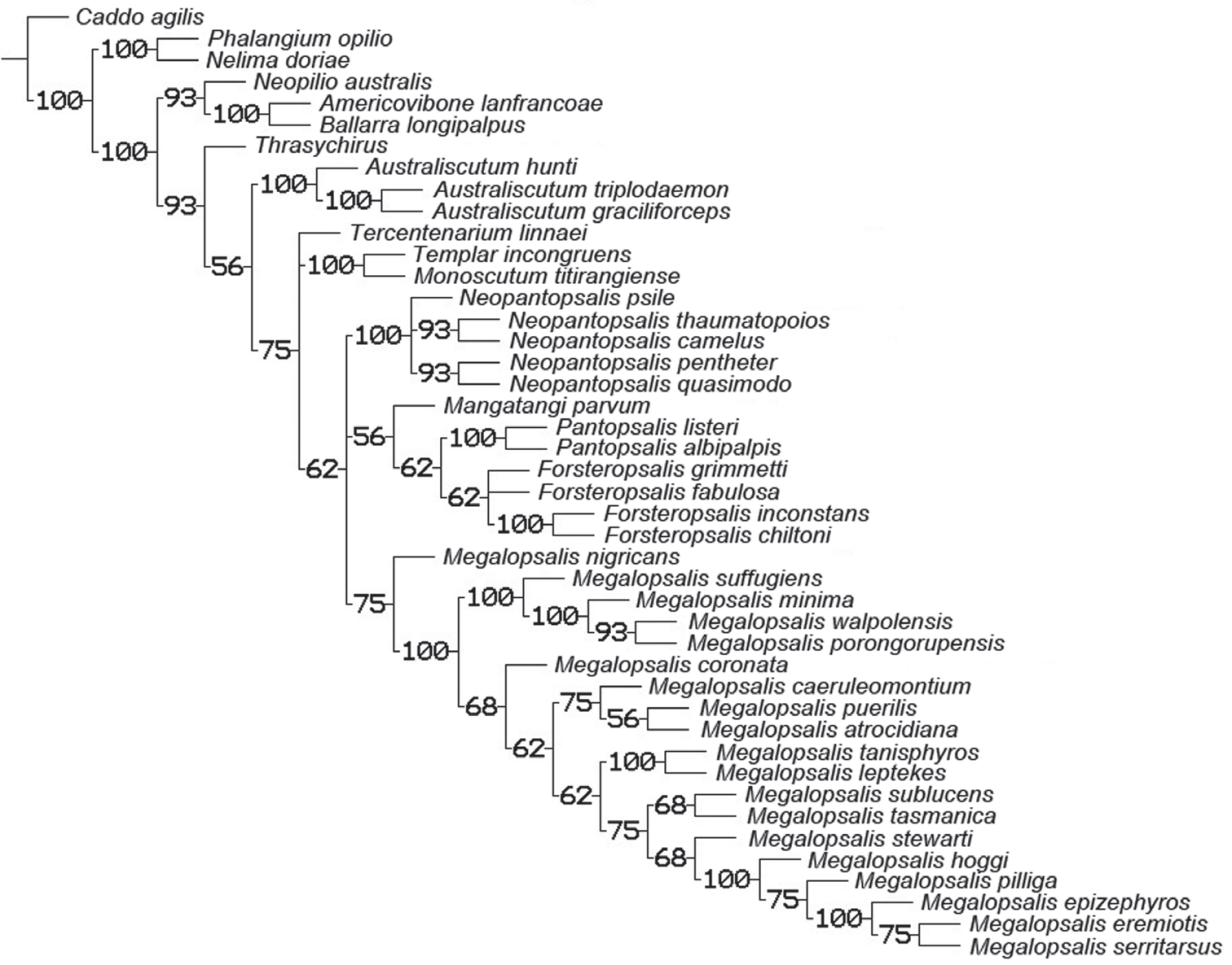
50% majority rule consensus of all trees resulting from all analyses. Numbers on branches indicate percentage of trees in which the given clade was recovered.

**Figure 25.**
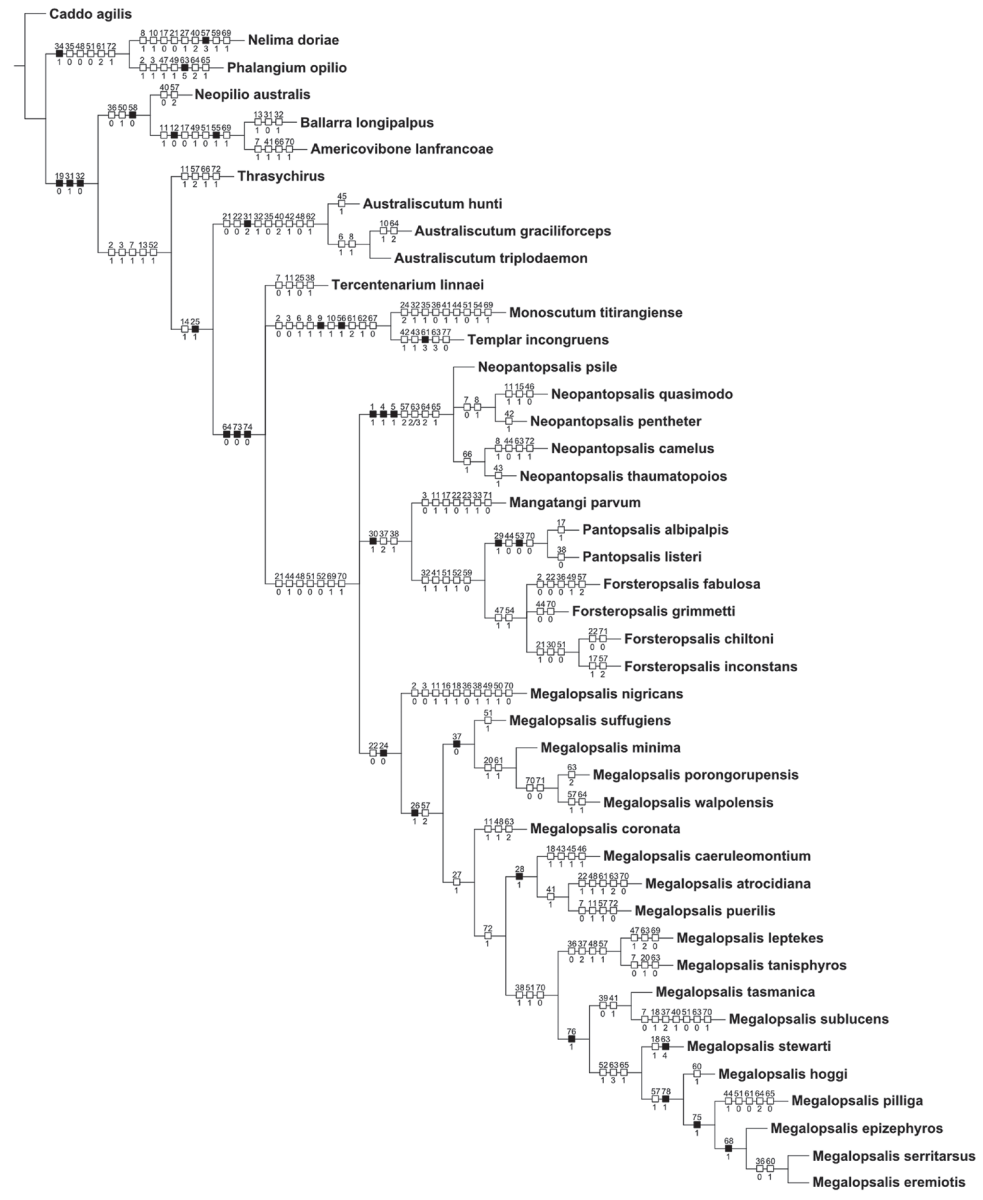
Character distribution mapped onto tree topology shown in Figure 24. Numbers above squares indicate character, numbers below squares indicate character state. Filled squares represent unique apomorphies, hollow squares represent homoplasious apomorphies.

A second round of analyses was also conducted with multistate characters treated as unordered. When all characters were given equal weight, this analysis produced two equally supported trees of 309 steps (CI = 0.301, RI = 0.633), the consensus of which is shown in Fig. 21. Though Neopilionidae was recovered as monophyletic in both trees, Enantiobuninae was only recovered in one tree, in which *Neopilio australis* and Ballarrinae were sister taxa as in the analyses with ordered characters. In the other best tree, *Neopilio australis* alone was sister to the remaining Neopilionidae, while *Tercentenarium linnaei* was placed outside a clade of Ballarrinae and the remaining Enantiobuninae. All implied weights analyses with unordered characters recovered a single best tree in which both Neopilionidae and Enantiobuninae were monophyletic. Figure 22 shows the majority-rule consensus for all analyses with unordered characters, with support for selected clades under individual weighting schemes shown in Table 2. Figure 23 shows the strict consensus of clades recovered under all analytical conditions, with a total majority-rule consensus shown in Fig. 24. Figure 25 shows the distribution of apomorphies when mapped onto the consensus tree in Fig. 24; some of these apomorphies are discussed below.

**Table 2.** Plot of recovery for selected clades under varying weight analyses with unordered characters. Grey fill indicates monophyly; black fill indicates monophyly in only one of two best trees; white fill indicates para- or polyphyly. Abbreviations as for Table 1.<br/>

*k* value	0.0	1.0	2.0	3.0	4.0	5.0	6.0
Neopilionidae							
Enantiobuninae							
Australasian Enantiobuninae							
*Australiscutum*							
*Mang*. + *Pant*. + *Forst*.							
*Pantopsalis* + *Forsteropsalis*							
*Pantopsalis*							
*Forsteropsalis*							
*Neopantopsalis*							
*Megalopsalis*							
*Megalopsalis* excl. *M. nigricans*							
*M. minima*-group							
*M. serritarsus*-group							

Neopilionidae is potentially supported by the absence of setae on the glans itself (char. 19), and by the presence of a longitudinal banner on the stylus (char. 31: lost within Ballarrinae, unknown for *Thrasychirus*). Reduction of the number of seminal receptacles to two (char. 32) is also mapped as a potential synapomorphy, but this character is reversed in *Australiscutum* and *Pantopsalis* + *Forsteropsalis*. Within Neopilionidae, most analyses supported a basal split between Enantiobuninae on one side and *Neopilio* + Ballarrinae on the other. The relationship between the latter two taxa is supported by one unique synapomorphy, the reduction in the pedipalpal claw (char. 58; Hunt and Cokendolpher 1991). None of the potential synapomorphies of Enantiobuninae are unique to that clade; they include the presence of large oblong ozopores on raised lateral lobes (chars 2, 3; reversed in *Megalopsalis nigricans*) and the presence of lateral processes behind the shaft-glans junction on the penis (char. 15; possibly homologous with the barbed process found in Australian Ballarrinae). Other potential synapomorphies, the presence of a distinct space between the ocularium and mesopeltidium (char. 7) and the presence of a pedipalpal patellar apophysis (char. 52) are both commonly reversed within Enantiobuninae.

The current analysis is, admittedly, limited in its ability to test neopilionid monophyly by the low number of outgroup taxa included. Members of all other families of Phalangioidea possess setae on the glans (Cokendolpher et al. 2007), as do males of *Caddo agilis* (Gruber, 1974). Males of Caddidae have been recorded on only rare occasions; other than *Caddo agilis*, males of Acropsopilioninae have genitalia that are highly modified relative to other Eupnoi (Shear 1975), making homologisation difficult. The lateral processes on the penis of Enantiobuninae (bristles or bristle groups) differ from the penile setae of other Palpatores in lacking a basal socket (Taylor 2011). Hedin et al. (2012) recovered a relationship between *Thrasychirus* and ‘*Megalopsalis*’ (probably *Forsteropsalis*, as the original specimen was collected in New Zealand) under most analytical conditions in a molecular phylogeny focused on Sclerosomatidae, but did not include *Neopilio* or Ballarrinae.

Many of the relationships within the Enantiobuninae are sensitive to weighting regimes, but the results are more stable than those found by Taylor (2011). Major clades supported by all analyses include *Australiscutum*, a clade of *Templar* + *Monoscutum* (henceforth referred to as the ‘*Monoscutum* clade’), *Pantopsalis*, *Neopantopsalis*, and a clade containing all species assigned herein to *Megalopsalis* except *Megalopsalis nigricans*. Implied weights analyses consistently placed *Thrasychirus* and *Australiscutum* outside a clade of the remaining Enantiobuninae; *Australiscutum* was sister taxon to the remaining Australasian Enantiobuninae when multistate characters were ordered, but was placed as sister to *Thrasychirus* in some analyses with multistate characters unordered. The equal weights analyses, in contrast, did not support monophyly of the Australasian Enantiobuninae. When multistate characters were ordered, a clade of *Tercentenarium* + the *Monoscutum* clade was positioned basalmost within the Enantiobuninae. When multistate characters were unordered, the equal weights analysis placed *Thrasychirus* and *Australiscutum* as a clade in a more nested position close to *Pantopsalis*, similar to the results found by Taylor (2011). However, as noted by Taylor (2011), *Thrasychirus* and *Australiscutum* differ from the remaining Enantiobuninae in their retention of a plesiomorphic mobile junction between the basitarsus and distitarsus (char. 73), and such a nested position would require that this character be reversed in these two taxa. Separation of *Thrasychirus* from the Australasian Enantiobuninae also aligns with the presence of bristle groups rather than isolated bristles at the shaft-glans junction in the latter (char. 14). Within *Australiscutum*, all analyses agree with Taylor (2011) in placing *Australiscutum triplodaemon* and *Australiscutum graciliforceps* closer to each other than *Australiscutum hunti*.

For the most part, the remaining relationships between genera in Enantiobuninae were not robust to analytical conditions. Analyses with multistate characters ordered supported a relationship between *Tercentenarium* and the *Monoscutum* clade, but this was not supported when multistate characters were unordered. Most analyses placed these taxa towards the base of the enantiobunines, which accords with their morphological distinctiveness. The New Zealand *Templar incongruens* and *Monoscutum titirangiense* (together with *Acihasta salebrosa*, not included in the current analysis but expected to be closely related to these two taxa) are short-legged, heavily sclerotised species previously classified as their own subfamily Monoscutinae (Forster 1948, Crawford 1992, Taylor 2008a). *Tercentenarium linnaei* possesses a number of features unique within the Neopilionidae, including a unilateral process on the left side of the penis at the shaft-glans junction, and a ‘keyhole’ emargination on the anterior margin of the female genital operculum (Taylor 2008b, 2011).

Implied weights analyses supported a relationship between the New Zealand genera *Pantopsalis* and *Forsteropsalis* as found by Taylor (2011), though this clade was not recovered in equal weights analyses. The genus *Forsteropsalis* formed a paraphyletic series in the equal weights analyses, but was returned to monophyly in implied weights analyses at *k* values of 2.0 and above. Implied weights analyses at a *k* value of 1.0 supported a clade of the New Zealand genera *Forsteropsalis*, *Pantopsalis* and *Mangatangi*, but *Pantopsalis* and *Mangatangi* were both nested within *Forsteropsalis*. *Forsteropsalis* species share two distinctive characters within the Enantiobuninae, a small triangular pedipalpal patellar apophysis (except in the female only of *Forsteropsalis grimmetti*) and an array of blunt denticles on the inner margin of the pedipalpal coxa (Taylor 2011). Taylor (2013) suggested that the newly described *Mangatangi parvum* might be the sister taxon to *Pantopsalis* + *Forsteropsalis*, but its position was variable depending on analysis conditions. At higher *k* values with multistate characters ordered, it was placed as sister to the eastern Australian genus *Neopantopsalis*.

*Neopantopsalis* was supported as monophyletic in all analyses, though its internal topology was sensitive to analysis conditions. Varying analyses placed it closer to either *Megalopsalis* or *Pantopsalis* + *Forsteropsalis*.

### Phylogeny of *Megalopsalis*

As found by Taylor (2011), the genus *Spinicrus* as hitherto recognised (Forster 1949, Hickman 1957, Kauri 1954) was not monophyletic, even with the segregation of *Neopantopsalis* (Taylor & Hunt, 2009). Instead, ‘*Spinicrus*’ species were placed as paraphyletic to *Megalopsalis* in sense of Taylor (2011). Members of *Megalopsalis* and *Spinicrus* are united by penis morphology, possessing a relatively short, broad, shallow glans that is more or less subtriangular in ventral view (char. 24). With the exception of ‘*Spinicrus*’ *nigricans*, the glans is also dorsally inflated near the shaft-glans junction, becoming distally narrower in lateral view, giving the glans a ‘bellied’ profile (char. 26; Figs 3e, 4e). In members of other enantiobunine genera, the glans is relatively much longer and/or deeper (Taylor 2004, 2008a, 2008b, 2009, 2011, 2013, Taylor and Hunt 2009).

*Spinicrus* and *Megalopsalis* were distinguished by the presence in the latter of a lateral apophysis on the patella of the pedipalp (Forster 1949). However, this feature is not unique to *Megalopsalis*, being also found in the *Monoscutum* clade, *Forsteropsalis* and females of *Pantopsalis*, and has probably appeared within the Enantiobuninae on multiple occasions. Even within the clade of *Spinicrus* and *Megalopsalis*, the presence of a pedipalpal apophysis may be homoplasious. While species with this character form a single clade in the equal weights analyses, the implied weights analyses all separate the *Megalopsalis leptekes*-groupfrom the *Megalopsalis serritarsus*-group (see below for group definitions), placing the latter as sister to ‘*Spinicrus*’ *stewarti*. As the distinction between *Megalopsalis* and *Spinicrus* thus appears less reliable phylogenetically than the synapomorphies connecting them, the two genera are here synonymised. *Hypomegalopsalis tanisphyros*, described by Taylor (2011) in its own genus owing to its then phylogenetically uncertain position, shares these synapomorphies and is also placed within *Megalopsalis*. In this broadened sense, *Megalopsalis* admittedly encompasses a higher morphological diversity than most other genera in Enantiobuninae (with the possible exception of *Forsteropsalis*: Taylor 2011). Nevertheless, a broadened *Megalopsalis* represents a far more practical solution to the apparent paraphyly of ‘*Spinicrus*’ than division of the latter between a number of small genera that would have to be distinguished by relatively small-scale and difficult characters.

For the most part, this expanded *Megalopsalis* forms a distinct clade in all analyses. The only exception is *Megalopsalis nigricans*, which is placed as the basalmost species of *Megalopsalis* in the implied weights analyses but does not form a clade with *Megalopsalis* in the equal weights analyses. The glans of *Megalopsalis nigricans* lacks the bellied profile of other *Megalopsalis* species, and is less regularly subtriangular than most other species. *Megalopsalis nigricans* also possesses a distinctly short and broad shaft to the penis compared to the relatively elongate and narrow shaft of other *Megalopsalis* species (Fig. 14b). Nevertheless, *Megalopsalis nigricans* is more similar in genital morphology to *Megalopsalis* speciesthan to other Enantiobuninae. Compared to other species of Enantiobuninae, *Megalopsalis nigricans* is a particularly small taxon with reduced armature, and it is possible that its position is being distorted in the equal weights analyses by homoplasy with similarly small-bodied taxa such as *Mangatangi parvum*. Rather than creating a new monotypic genus for this species, *Megalopsalis nigricans* is here provisionally assigned to *Megalopsalis*.

Within the clade of *Megalopsalis* species other than *Megalopsalis nigricans*, a small number of clades are recovered in all analyses, treated here as the *Megalopsalis leptekes*-, *Megalopsalis minima*- and *Megalopsalis serritarsus*-species groups. The *Megalopsalis leptekes*-group includes the two Western Australian species *Megalopsalis leptekes* and *Megalopsalis tanisphyros*. Though a number of potential synapomorphies are identified for these two species in Fig. 25, none are unique to this clade; these include the presence of an elongate, hypersetose pedipalpal patellar apophysis, and an opisthosomal spiracle with relatively long, non-reticulate covering spines extending across the entire breadth of the spiracle rather than only partway as in other *Megalopsalis* species (Taylor 2011).

The species of the *Megalopsalis minima*-group are similarly Western Australian in distribution, comprising *Megalopsalis minima*, *Megalopsalis porongorupensis*, *Megalopsalis suffugiens* and *Megalopsalis walpolensis*. Members of this group are also united by spiracle morphology, with the covering spines being mostly lost (char. 37) though remnant lace tubercles are retained except in *Megalopsalis suffugiens* (in which they have been reduced to a patch of reticulate ornamentation only). All analyses also agree in placing *Megalopsalis suffugiens*, found in the Nullarbor in south-east Western Australia, as the sister taxon to the remaining species found in the south-west corner of Western Australia.

The *Megalopsalis serritarsus*-group corresponds to the ‘core *Megalopsalis* clade’ of Taylor (2011). Members of this group possess an elongate pedipalpal patellar apophysis, and males have specialised brush-like setae in a ventral double row on basally inflated distitarsi III and IV (Taylor 2011). With the exception of *Megalopsalis hoggi*, males of the *Megalopsalis serritarsus*-group also have modified second tarsi with ventrodistal swellings on the basal pseudosegments (Taylor 2011), and the majority of analyses agree with Taylor (2011) in placing *Megalopsalis hoggi* as sister to the remaining species. The brush-like setae on distitarsi III and IV are shared with four further species: *Megalopsalis stewarti*, *Megalopsalis sublucens*, *Megalopsalis tasmanica* and *Megalopsalis thryptica*. For the most part, these species do not also have the distitarsi inflated as in the *Megalopsalis serritarsus*-group, though *Megalopsalis thryptica* has distitarsus IV only basally inflated (Hickman 1957; *Megalopsalis thryptica* was omitted from the phylogenetic analysis owing to the unavailability of specimens). In the implied weights analyses, all species with brush-like setae are placed in a single clade; as this character is not found elsewhere in the Enantiobuninae, this result seems more credible than that of the equal weights analysis which unites the *Megalopsalis serritarsus*-group with the *Megalopsalis leptekes*-group on the basis of the probably homoplasious pedipalpal patellar apophysis.

Relationships between the remaining species of *Megalopsalis* are not consistently recovered by all analyses, and they are left unplaced in species groups. Clades recovered by implied weights analyses but not by the equal weights analyses include a sister relationship between the Tasmanian species *Megalopsalis tasmanica* and *Megalopsalis sublucens*, and a clade including *Megalopsalis atrocidiana*, *Megalopsalis caeruleomontium* and *Megalopsalis puerilis*. The latter three species share a genital morphology with the sides of the glans closer to parallel in ventral view than in other *Megalopsalis* species (char. 28).

### Biogeography and male variation

Though neither Australian nor New Zealand Enantiobuninae are identified as monophyletic, there is an overall separation between the fauna of the two land masses. New Zealand taxa may belong to as few as two separate clades (depending on the position of *Mangatangi parvum*). A relatively low level of interchange is also indicated between the western and eastern sides of the Australian continent, with the Western Australian species of *Megalopsalis* mostly assignable to the endemic clades of the *Megalopsalis minima*- and *Megalopsalis leptekes*-groups. *Megalopsalis epizephyros* is a member of the otherwise eastern Australian *Megalopsalis serritarsus*-group and may represent a more recent immigration; it is notable in this regard that the only Enantiobuninae known to date from South Australia are representatives of the *Megalopsalis serritarsus*-group (Forster 1949, Taylor 2011). In general, members of the *Megalopsalis leptekes*- and *Megalopsalis serritarsus*-groups tend to be found in more inland, and presumably drier, localities than other *Megalopsalis* species (Taylor 2011).

The feature of Australasian Enantiobuninae that has perhaps caused the most comment is the presence in males of most species of greatly enlarged chelicerae relative to the females. Though this has been cited as a diagnostic characteristic of the group (e.g. Dunlop and Mammitzsch 2010), many species also exhibit minor males with chelicerae that are less exaggerated than those of majors (Taylor and Hunt 2009). For a number of species of *Megalopsalis* (*Megalopsalis coronata*, *Megalopsalis puerilis*, *Megalopsalis sublucens*, *Megalopsalis tanisphyros* and *Megalopsalis walpolensis*), the males appear to lack enlarged chelicerae (Taylor 2011 and below), though the possibility cannot be excluded that major males of these species remain to be discovered. The ratios of major to minor males in a population may vary between species: in *Neopantopsalis* species, for instance, the majority of specimens in collections are majors, while in *Megalopsalis minima* and *Megalopsalis nigricans*, majors are relatively rare and greatly outnumbered by minors (personal observations).

Variation in male cheliceral development has been identified for species of *Megalopsalis*, *Neopantopsalis* and *Pantopsalis* (Taylor 2004, 2013, Taylor and Hunt 2009; as noted by Taylor 2013, the ‘effeminate’ males described by Forster 1964 and Taylor 2004 for certain *Pantopsalis* species probably represent young specimens that have yet to complete cuticular hardening). Specimens of *Forsteropsalis chiltoni* vary in the degree of inflation of the second cheliceral segment, though they are not divisible between discrete morphs (Taylor 2011). In *Pantopsalis*, broad- and slender-chelicerate males differ in cheliceral length and inflation of the second segment, but males otherwise do not differ significantly in body size (Taylor 2004). In *Neopantopsalis* and *Megalopsalis*, major and minor males distinctly differ in body size as well as cheliceral size. It seems likely that discrete male dimorphism has developed independently in *Pantopsalis* versus *Neopantopsalis* + *Megalopsalis*, though it is possible that a more general variability as seen in *Forsteropsalis chiltoni* may be ancestral for the larger clade containing all three genera.

Within the Australian taxa, discrete major and minor morphs (i.e. without intermediate-sized individuals) are identifiable in *Neopantopsalis* species, *Megalopsalis minima* and *Megalopsalis nigricans*. However, *Megalopsalis caeruleomontium*, *Megalopsalis porongorupensis* and *Megalopsalis stewarti* exhibit more continual variation without clearly discrete morphs (variation is also present in *Megalopsalis suffugiens*, but sample size is not large enough to identify whether it is discrete or continuous). Most other *Megalopsalis* species are known from too few specimens to discount the possibility of male variation, with the possible exception of *Megalopsalis epizephyros* (Taylor 2011). It seems likely that at least some degree of male variation is ancestral for *Megalopsalis*, though we cannot say whether it was discrete or continuous.

## Supplementary Material

XML Treatment for Megalopsalis

XML Treatment for Megalopsalis minima

XML Treatment for Megalopsalis porongorupensis

XML Treatment for Megalopsalis suffugiens

XML Treatment for Megalopsalis walpolensis

XML Treatment for Megalopsalis atrocidiana

XML Treatment for Megalopsalis caeruleomontium

XML Treatment for Megalopsalis coronata

XML Treatment for Megalopsalis nigricans

XML Treatment for Megalopsalis puerilis

XML Treatment for Megalopsalis stewarti

XML Treatment for Megalopsalis sublucens

## Appendix

### Character state matrix for phylogenetic analysis

Caddo_agilis

0 0 0 0 0 0 0 0 0 0 0 1 0 0 ? ? 1 0 1 ? ? 1 1 1 0 0 0 0 0 0 0 1 0 0 1 1 ? ? ? 0 0 0 0 0 0 0 0 1 0 0 1 0 ? ? 0 0 0 1 0 0 0 0 ? ? 0 0 0 ? 0 0 1 0 1 1 ? ? 0 ?

Nelima_doriae

0 0 0 0 0 0 0 1 0 1 0 1 0 0 ? ? 0 0 1 0 0 1 0 2 0 0 1 0 0 0 0 1 0 1 0 ? ? ? ? 2 0 0 0 0 0 0 0 0 0 0 0 0 ? ? 0 0 3 1 1 0 2 0 2 1 0 0 1 0 1 0 1 1 1 1 0 0 1 0

Phalangium_opilio

0 1 1 0 0 0 0 0 0 0 0 1 0 0 ? ? 1 0 1 0 1 1 1 2 0 0 0 0 0 0 0 ? ? 1 0 ? ? ? ? 1 0 0 0 0 0 0 1 0 1 0 0 ? ? ? 0 0 1 1 0 0 2 0 5 2 1 0 1 0 0 0 1 1 1 1 0 0 1 0

Neopilio_australis

0 ? ? 0 0 0 0 0 0 0 0 1 0 0 ? ? 1 0 0 ? ? 1 ? 2 0 0 0 0 0 ? 1 0 1 0 2 0 ? ? ? 0 0 0 0 0 0 ? ? 1 0 1 1 0 ? ? 0 0 2 0 0 0 0 0 0 ? 0 0 1 ? 0 0 ? ? ? ? 0 0 ? 0

Ballarra_longipalpus

0 0 0 0 0 0 0 0 0 0 1 0 1 1 ? 1 0 0 0 0 1 1 0 2 0 0 0 0 0 0 0 1 1 0 0 0 ? ? ? 2 0 0 0 0 0 0 0 1 1 1 0 0 ? ? 1 0 0 0 ? 0 0 0 0 ? 0 0 1 0 1 0 1 0 1 1 0 0 1 0

Americovibone_lanfrancoae

0 0 0 0 0 0 1 0 0 0 1 0 0 ? ? ? 0 0 0 ? ? 1 0 2 0 0 0 0 0 0 1 0 ? ? ? ? ? ? ? ? 1 0 0 0 0 0 0 1 1 1 0 0 ? ? 1 0 ? 0 ? 0 0 0 0 ? ? 1 1 0 1 1 ? ? ? ? ? ? ? ?

Thrasychirus

0 1 1 0 0 0 1 ? 0 0 1 ? 1 0 ? ? ? 0 0 ? ? 1 ? 1 0 0 0 0 ? ? ? 0 1 0 2 1 2 0 0 1 1 0 1 ? ? 0 ? 1 0 0 1 1 ? ? 0 0 2 1 1 ? ? 0 ? ? ? 1 1 ? ? ? 1 1 1 1 ? ? 1 ?

Australiscutum_hunti

0 1 1 0 0 0 1 0 0 0 0 1 1 1 1 0 1 0 0 0 0 0 1 1 1 0 0 0 0 0 2 1 0 0 0 ? ? ? ? 2 1 1 1 0 1 0 0 0 0 0 1 1 ? ? 0 0 1 1 1 0 0 1 0 ? 0 0 1 0 0 0 0 ? 1 1 0 0 1 0

Australiscutum_graciliforceps

0 1 1 0 0 1 1 1 0 1 0 1 1 1 1 0 1 0 0 0 0 0 1 1 1 0 0 0 0 0 2 1 0 0 0 ? ? ? ? 2 1 1 1 0 0 0 0 0 0 0 1 1 1 0 0 0 1 1 1 0 0 1 1 2 0 0 1 0 0 0 0 ? 1 1 0 0 1 0

Australiscutum_triplodaemon

0 1 1 0 0 1 1 1 0 0 0 1 1 1 1 0 1 0 0 0 0 0 1 1 1 0 0 0 0 0 2 ? 0 0 0 ? ? ? ? 2 ? 1 1 0 [01]0 0 0 0 0 1 ? 1 0 0 0 1 1 1 0 0 1 1 1 0 0 1 0 0 0 0 ? 1 1 0 0 1 0

Monoscutum_titirangiense

0 0 0 0 0 1 ? 1 1 1 0 1 1 1 0 0 0 0 0 0 1 1 0 2 1 0 0 0 0 0 1 1 0 0 1 0 ? ? ? 1 1 0 0 1 0 0 0 1 0 0 0 2 1 1 0 1 1 1 0 0 2 1 0 ? 0 0 0 0 1 0 1 0 0 0 0 0 1 0

Templar_incongruens

0 0 0 0 0 1 ? 1 1 1 ? 1 1 1 0 ? 0 0 0 ? ? 1 0 1 1 0 0 0 0 ? 1 0 0 0 2 1 1 0 ? 1 0 1 1 0 0 0 0 ? 0 0 1 2 1 0 0 1 1 1 0 0 3 1 3 0 0 0 0 0 0 0 0 ? 0 0 0 0 0 0

Tercentenarium_linnaei

0 1 1 0 0 0 0 0 0 0 1 1 1 1 0 0 1 0 0 0 1 1 1 1 0 0 0 0 0 0 1 0 0 0 2 1 1 1 1 0 0 0 0 0 0 0 0 1 0 0 1 2 1 0 0 0 1 1 0 0 0 0 0 ? 0 0 1 0 0 0 0 ? 0 0 0 0 1 0

Forsteropsalis_chiltoni

0 1 1 0 0 0 1 0 0 0 0 1 1 1 0 0 0 0 0 0 1 0 0 1 1 0 0 0 0 0 1 1 0 0 2 1 2 1 0 1 ? 0 0 1 1 1 1 0 0 0 0 1 1 1 0 0 1 1 0 0 0 0 1 0 0 0 1 0 1 1 0 1 0 0 0 0 1 0

Forsteropsalis_fabulosa

0 0 1 0 0 0 1 0 0 0 0 1 1 1 0 0 0 0 0 0 0 0 0 1 1 0 0 0 0 1 1 ? 0 0 2 0 2 1 0 1 1 0 1 1 1 1 1 0 1 0 1 1 1 1 0 0 2 1 0 0 0 0 1 0 0 0 1 0 1 1[01] 1 0 0 0 0 1 0

Forsteropsalis_grimmetti

0 1 1 0 0 0 1 0 0 0 0 1 1 1 0 0 0 0 0 0 0 1 0 1 1 0 0 0 0 1 1 1 0 0 2 1 2 1 0 1 1 0 1 0 1 1 1 0 0 0 1 1 1 [01]0 0 1 1 0 0 0 0 1 0 0 0 1 0 1 0 1 1 0 0 0 0 1 0

Forsteropsalis_inconstans

0 1 1 0 0 0 1 0 0 0 0 1 1 1 0 0 1 0 0 0 1 1 0 1 1 0 0 0 0 0 1 ? 0 0 2 1 2 ? 0 1 0 0 0 1 1 1 1 0 0 0 0 1 1 1 0 0 2 1 0 0 0 0 1 0 0 0 1 0 1 1 1 1 0 0 0 0 1 0

Pantopsalis_albipalpis

0 1 1 0 0 0 1 0 0 0 0 1 1 1 0 0 1 0 0 0 0 1 0 1 1 0 0 0 1 1 1 1 0 0 2 1 2 1 1 0 1 0[01]0 0 1 0 0 0 0 1 1 0 0 0 0 1 1 0 0 0 0 1 0 0 0 1 0 1 0 1 1 0 0 0 0 1 0

Pantopsalis_listeri

0 1 1 0 0 0 1 0 0 0 0 1 1 1 0 0 0 0 0 0 0 1 0 1 1 0 0 0 1 1 1 1 0 0 2 1 2 0 0 1 1 0 0 0 0 1 0 0 0 0 1 1 0 0 0 0 1 1 0 0 0 0 1 0 0 0 1 0 1 0 1 1 0 0 0 0 1 0

Mangatangi_parvum

0 1 0 0 0 0 1 0 0 0 1 1 1 1 0 ? 1 0 0 ? ? 0 1 1 1 0 0 0 0 1 1 0 1 0 2 ? 2 1 1 0 0 0 1 1 1 1 0 0 0 0 0 0 ? ? 0 0 1 1 1 0 0 0 0 ? ? ? 1 ? ? 1 0 ? 0 0 ? 0 1 0

Neopantopsalis_quasimodo

1 1 1 1 1 0 0 1 0 0 1 1 1 1 1 0 0 0 0 0 0 1 0 1 1 0 0 0 0 0 1 ? 0 0 2 0 1 0 ? 0 ? 0 0 1 1 0 0 0 0 0 0 ? ? ? 0 0 2 1 1 0 1 0 3 2 1 0 1 0 1 1 1 0 0 0 0 0 1 0

Neopantopsalis_camelus

1 1 1 1 1 1 1 1 0 0 0 1 1 1 0 ? 0 0 0 ? ? 1 0 1 1 0 0 0 0 0 1 ? 0 0 2 ? 1 1 ? ? ? 0 0 0 1 1 0 ? 0 0 0 ? ? ? 0 0 2 1 1 0 0 0 1 2 1 1 1 0 1 1 1 1 0 0 0 0 1 0

Neopantopsalis_pentheter

1 1 1 1 1 0 0 1 0 0 0 1 1 1 0 0 0 0 0 0 0 1 0 1 1 0 0 0 0 0 1 0 0 0 2 1 1 0 1 0 0 1 0 1 1 1 0 0 0 0 0 0 ? ? 0 0 2 1 1 0 1 0 3 2 1 0 1 0 1 1 1 0 0 0 0 0 1 0

Neopantopsalis_psile

1 1 1 1 1 1 1 0 0 0 0 1 1 1 0 0 0 0 0 0 0 1 0 1 1 0 0 0 0 0 1 0 0 0 2 0 1 0 1 0 0 0 0 1 1 1 0 0 0 0 0 0 ? ? 0 0 2 1 1 0 1 0 3 2 1 0 1 0 1 1 1 0 0 0 0 0 1 0

Neopantopsalis_thaumatopoios

1 1 1 1 1 1 1 0 0 0 0 1 1 1 0 ? 0 0 0 ? ? 1 0 1 1 0 0 0 0 0 1 ? ? ? ? ? ? ? ? ? ? 0 1 1 1 1 0 ? 0 0 0 ? ? ? 0 0 2 1 1 0 0 0 2 2 1 1 1 0 1 ? 1 0 0 0 0 ? 1 ?

Megalopsalis_serritarsus

0 1 1 0 0 0 1 0 0 0 0 1 1 1 0 ? 0 0 0 ? ? 0 0 0 1 1 1 0 0 0 1 ? 0 0 2 0 1 1 1 0 ? 0 0 0 0 0 0 1 0 0 1 ? 1 0 0 0 1 1 1 1 1 0 3 1 1 0 1 1 1 0 0 ? 0 0 1 1 1 1

Megalopsalis_epizephyros

0 1 1 0 0 0 1 0 0 0 0 1 1 1 0 0 0 0 0 0 0 0 0 0 1 1 1 0 0 0 1 0 0 0 2 1 1 0 1 0 0 0 0 0 0 0 0 1 0 0 1 2 1 0 0 0 1 1 1 0 1 0 3 1 1 0 1 1 1 0 0 ? 0 0 1 1 1 1

Megalopsalis_eremiotis

0 1 1 0 0 0 1 0 0 0 0 1 1 1 0 0 0 0 0 1 0 0 0 0 1 1 1 0 0 0 1 0 0 0 2 0 1 0 1 0 0 0 0 0 0 0 0 1 0 0 1 2 1 0 0 0 1 1 1 1 1 0 3 1 1 0 1 1 1 0 0 ? 0 0 1 1 1 1

Megalopsalis_hoggi

0 1 1 0 0 0 1 0 0 0 0 1 1 1 0 ? 0 0 0 ? ? 0 0 0 1 1 1 0 0 0 1 ? ? ? ? ? ? ? ? ? ? 0 0 0 0 0 0 ? 0 0 1 ? 1 0 0 0 1 1 1 1 1 0 3 1 1 0 1 0 1 0 0 ? 0 0 0 1 1 1

Megalopsalis_leptekes

0 1 1 0 0 0 1 0 0 0 0 1 1 1 0 0 0 0 0 0 0 0 0 0 1 1 1 0 0 0 1 ? 0 0 2 0 2 1 ? 0 ? 0 0 0 0 0 1 1 0 0 1 ? 1 0 0 0 1 1 1 0 0 0 2 1 0 0 1 0 0 0 0 ? 0 0 0 0 1 0

Megalopsalis_pilliga

0 1 1 0 0 0 1 0 0 0 0 1 1 1 0 0 0 0 0 0 0 0 0 0 1 1 1 0 0 0 1 ? ? ? ? ? ? ? ? ? ? 0 0 1 0 0 0 1 0 0 0 ? 1 0 0 0 1 1 1 0 0 0 3 2 0 0 1 0 1 0 0 ? 0 0 1 1 1 1

Megalopsalis_tanisphyros

0 1 1 0 0 0 0 0 0 0 0 1 1 1 0 0 0 0 0 1 0 0 0 0 1 1 1 0 0 0 1 ? 0 0 2 0 2 1 1 0 0 0 0 1 0 0 0 1 0 0 1 ? 1 0 0 0 1 1 1 0 0 0 0 ? 0 0 1 0 1 0 0 ? 0 0 0 0 1 0

Megalopsalis_tasmanica

0 1 1 0 0 0 1 0 0 0 0 1 1 1 0 0 0 0 0 1 0 0 0 0 1 1 1 0 0 0 1 1 0 0 2 1 1 1 0 0 1 0 0 0 0 0 0 0 0 0 1 0 ? ? 0 0 2 1 1 0 1 0 1 1 0 0 1 0 1 0 1 1 0 0 0 1 1 0

Megalopsalis_minima

0 1 1 0 0 0 0 0 0 0 0 1 1 1 0 0 0 0 0 1 0 0 0 0 1 1 0 0 0 0 1 ? 0 0 2 ? 0 ? 1 0 ? 0 0 1 0 0 0 0 0 0 0 ? ? ? 0 0 2 1 1 0 1 0[12]0 0 0 1 0 1 1 1 0 0 0 0 0 1 0

Megalopsalis_nigricans

0 0 0 0 0 0 1 0 0 0 1 1 1 1 0 1 0 1 0 0 0 0 0 0 1 0 0 0 0 0 1 0 0 0 2 0 1 1 1 0 0 0 0 1 0 0 0 0 1 1 0 0 ? ? 0 0 1 1 1 0 0 0 0 ? 0 0 1 0 1 0 1 0 0 0 0 0 1 0

Megalopsalis_porongorupensis

0 1 1 0 0 0 0 0 0 0 0 1 1 1 0 0 0 0 0 1 0 0 0 0 1 1 0 0 0 0 1 ? 0 0 2 ? 0 ? 1 0 ? 0 0 0 0 0 0 0 0 0 0 ? ? ? 0 0 2 1 1 0 1 0 2 0 0 0 1 0 1 0 0 ? 0 0 0 0 1 0

Megalopsalis_stewarti

0 1 1 0 0 0 1 0 0 0 0 1 1 1 0 0 0 1 0 0 0 0 0 0 1 1 1 0 0 0 1 1 0 0 2 1 1 1 1 0 0 0 0 0 0 0 0 0 0 0 1 1 ? ? 0 0 2 1 1 0 1 0 4 1 1 0 1 0 1 0 1 1 0 0 0 1 1 0

Megalopsalis_sublucens

0 1 1 0 0 0 0 0 0 0 0 1 1 1 0 ? 0 1 ? ? ? 0 0 0 1 1 1 0 0 0 1 ? 0 0 2 ? 2 1 0 1 1 0 0 0 0 0 0 0 0 0 0 ? ? ? 0 0 2 1 1 0 0 0 0 ? 0 0 1 0 1 1 0 ? 0 0 0 1 1 0

Megalopsalis_suffugiens

0 1 1 0 0 0 0 0 0 0 0 1 1 1 0 0 0 0 0 0 0 0 0 0 1 1 0 0 0 0 1 1 0 0 2 ? 0 ? 1 0 0 0 0 0 0 0 0 0 0 0 1 0 ? ? 0 0 2 1 1 0 0 0 1 [01]0 0 1 0 1 1 1 0 0 0 0 0 1 0

Megalopsalis_walpolensis

0 1 1 0 0 0 0 0 0 0 0 1 1 1 0 0 0 0 0 1 0 0 0 0 1 1 0 0 0 0 1 ? 0 0 2 ? 0 ? 1 0 ? 0 0 0 0 0 0 0 0 0 0 ? ? ? 0 0 1 1 1 0 1 0 1 1 0 0 1 0 1 0 0 ? 0 0 0 0 1 0

Megalopsalis_atrocidiana

0 1 1 0 0 0 1 0 0 0 0 1 1 1 0 ? 0 0 ? ? ? 1 0 0 1 1 1 1 0 0 1 0 0 0 2 1 1 0 1 0 1 0 0 0 0 0 0 1 0 0 0 1 ? ? 0 0 2 1 1 0 1 0 2 1 0 0 1 0 1 0 1 1 0 0 0 0 1 0

Megalopsalis_coronata

0 1 1 0 0 0 0 0 0 0 1 1 1 1 0 0 0 0 0 0 0 0 0 0 1 1 1 0 0 0 1 ? 0 0 2 1 1 0 1 0 0 0 0 1 0 0 0 1 0 0 0 ? ? ? 0 0 2 1 1 0 0 0 2 0 0 0 1 0 1 1[01]0 0 0 0 0 1 0

Megalopsalis_puerilis

0 1 1 0 0 0 0 0 0 0 1 1 1 1 0 0 0 0 0 1 0 0 0 0 1 1 1 1 0 0 1 ? 0 0 2 1 1 0 1 0 1 0 0 0 0 0 0 0 0 0 0 ? ? ? 0 0 1 1 1 0 0 0 1 1 0 0 1 0 1 1 [01]0 0 0 0 0 1 0

Megalopsalis_caeruleomontium

0 1 1 0 0 0 1 0 0 0 0 1 1 1 0 0 0 1 0 0 0 0 0 0 1 1 1 1 0 0 1 ? 0 0 2 1 1 0 1 0 0 0 1 1 1 1 0 0 0 0 0 0 ? ? 0 0 2 1 1 0 0 0 1 0 0 0 1 0 1 1 1 1 0 0 0 0 1 0
